# Evaluating algorithmic approaches to rare disease case-finding: a retrospective validation study using electronic health records

**DOI:** 10.1186/s13023-026-04240-6

**Published:** 2026-02-04

**Authors:** Freya Boardman-Pretty, Jyothika Kumar, Calum Grant, Elena Marchini, William Evans, Lara Menzies, Rand Dubis, Amanda Worker, Elizabeth Varones, Alan Warren, Jack Sams, Daniel Ollerenshaw, Jez Stockdale, Hadley Mahon, Peter Fish

**Affiliations:** 1 Mendelian, The Trampery, Old Street, London, UK; 2 Health Innovation East, Shelford Bottom, Cambridge, UK; 3 Centre for Academic Primary Care, University of Nottingham, Nottingham, UK; 4 Yorkshire Regional Genetics Service, Leeds Teaching Hospitals NHS Trust, Chapel Allerton Hospital, Leeds, UK; 5 Department of Clinical Genetics, Great Ormond Street Hospital for Children NHS Foundation Trust, London, UK

**Keywords:** Rare disease, Diagnosis, Case-finding, Algorithm, Validation, EHR

## Abstract

**Background:**

Accurate and early diagnosis to optimise rare disease care is a global priority. With recent developments in artificial intelligence (AI)-based solutions, a promising area to improve rare disease diagnosis is the application of AI to routinely collected health care data captured in electronic health records (EHRs). MendelScan is a rare disease case-finding tool that analyses structured EHR data, using algorithms to identify patterns that are associated with an increased likelihood of the patient being affected by one of a number of rare diseases, in order to put such patients forward for further review.

**Methods:**

In this paper, we evaluated the performance of case-finding algorithms for 34 rare diseases within MendelScan, by performing a retrospective validation study using research EHR data. The primary objectives were to assess MendelScan’s ability to correctly identify cases versus controls, and to investigate other metrics indicating feasibility of large-scale deployment and time identified (flagged) relative to diagnosis. We measured algorithm performance by sensitivity, specificity, positive predictive value (PPV), and likelihood ratios.

**Results:**

Algorithm performance varied from metric to metric for the different algorithms. Sensitivity ranged from 0 to 100%, but majority were under 25% (median = 3.8% (IQR: 1.2–12.6%)) whereas specificity for most algorithms was above 99.995% (median = 99.9966% (IQR: 99.9925–99.9988)). Median PPV adjusted by literature prevalence was 3.1% (IQR: 0.7–14.6%) and by coded prevalence, 2.5% (IQR: 0.4–8.4). Median positive likelihood ratio was 1167 (IQR: 125–4006), reflecting a strong signal for disease presence, and median negative likelihood ratio was 0.96 (IQR: 0.87–0.99), reflecting limited clinical utility of a negative result.

**Conclusions:**

Our findings demonstrate the potential of using routinely collected EHR data to facilitate earlier diagnosis of rare diseases. Real world evaluations are required in order to fully ascertain the impact of such case-finding algorithms in assisting with the detection and diagnosis of patients with rare diseases.

**Supplementary Information:**

The online version contains supplementary material available at 10.1186/s13023-026-04240-6.

## Background

Rare diseases are individually rare but collectively common. With more than 7000 rare diseases, 1 in 17 people will be affected by a rare disease in their lifetime, with an estimated 400 million people living with a rare disease globally [1, 2]. Early and accurate diagnosis of rare diseases is a major challenge worldwide. Patients frequently experience diagnostic delays, misdiagnoses or no diagnosis at all, despite multiple primary care visits, specialist clinic reviews and investigations. This is referred to as the “diagnostic odyssey” [3, 4]. In the UK, studies have reported that the average diagnostic delay for a cohort of rare diseases patients was 5.6 years and that 50% of rare disease patients are never diagnosed [5, 6]. This under-diagnosis makes it particularly challenging to accurately quantify the burden of rare diseases. Therefore, their impact on healthcare systems is expected to be substantially under-estimated.

The need for an accurate and early diagnosis to optimise rare disease care is acknowledged globally, and is a priority of the 2021 UK Rare Diseases Framework [7, 8]. For most emerging rare disease therapies, early intervention is key for optimal response to treatment and clinical outcome. Advances in genomic technologies have enabled easier and more accurate molecular diagnoses; however, they require the correct patients to be advanced for such testing. Primary care clinicians play an important role in identifying patients who may have a rare disease and advancing them along the appropriate clinical pathway.

However, typically general practitioners have limited experience and knowledge of rare diseases and lack the tools to help them recognise a patient who may have a rare disease [9]. This challenge is frequently compounded by significant variability in disease features not only across diseases, but also how an individual disease may present from one patient to the next. Furthermore, there is often significant overlap in presentation with common diseases. Therefore, to help shorten the diagnostic odyssey there is a need to support clinicians to recognise uncommon patterns in clinical presentations and to consider rare diseases in their differential diagnoses [10].

With recent developments in artificial intelligence (AI)-based solutions, multiple tools have been developed which can aid in piecing together patients’ clinical signs and symptoms to highlight the possibility of a rare disease aetiology [11–13]. Such diagnostic or clinical decision support systems can be developed using different approaches and can help clinicians diagnose rare diseases. For example, previous research has shown that an AI database which calculates disease probabilities based on patient symptoms showed promising results in providing accurate rare disease diagnoses when tested on retrospective data [13].

A promising area to improve rare disease diagnosis is the application of AI to routinely collected health care data captured in electronic health records (EHRs). EHRs are a digital representation of a patient’s medical history, including diagnoses, clinical events, medications, referrals and test results. Much of this information is stored in a structured form, using standardised clinical vocabularies such as SNOMED-CT [14] or CTV3 (“Read”) [15] codes, with other information captured in an unstructured format such as notes and letters. Health records in the UK are now mostly recorded and held in an electronic format. The standardised format and large scale of structured EHR data provides a good basis for the application of AI-driven case-finding methods, to utilise the richness of a patient’s medical history and connect diagnoses and features that may have been viewed as independent previously.

Examples of such work include an algorithm that learns phenotypic patterns from confirmed common variable immunodeficiency syndrome cases and uses this knowledge to rank undiagnosed patients by likelihood of having the disease [16]. Although such developments are promising, there are many practical challenges in deploying these tools widely across healthcare systems. Although there are some “common” disease prediction tools deployed, such as for the electronic flagging of patients at risk of hepatitis C in primary and secondary care settings [17], there are not, to our knowledge, any rare disease clinical decision support tools adopted within the NHS.

Mendelian is a UK-based digital health company focused on the accurate and timely diagnosis of rare and hard to diagnose diseases. Mendelian have developed MendelScan, a rare disease case-finding tool that analyses structured EHR data, using algorithms to identify patterns that are associated with an increased likelihood of the patient being affected by one of a number of rare diseases. It is designed as a tool to screen and prioritise patients, not to make diagnoses. Patients identified (flagged) by these algorithms are surfaced for further clinical review by clinicians, who may then choose to initiate the appropriate diagnostic pathway. These algorithms are routinely edited to improve performance, and the metrics shown in this study represent the set of algorithms at one point in time, with performance expected to improve in future. Such case-finding tools can be deployed in primary care, particularly in the UK where primary care EHR coverage is ubiquitous. Primary care is a key bottleneck where early signs and symptoms associated with rare diseases can be missed and / or misidentified.

In this paper, we evaluate the performance of case-finding algorithms for 34 rare diseases by performing a retrospective validation study using research EHR data. The primary objectives were to (i) assess MendelScan’s ability to correctly identify cases and controls and (ii) that the cases are flagged in advance of their current diagnosis. This is part of a larger piece of work to assess MendelScan that includes assessments of clinical utility, cost effectiveness, and real world deployments, as guided by the NICE Evidence Standards Framework for digital health technologies [18].

## Methods

### Algorithm development

Due to the diverse nature of symptoms and presentations of rare diseases, we adopted a bespoke, disease-level approach. Each algorithm was developed to target a specific disease or clinical indication. Algorithms were prioritised based on the following criteria: (1) likelihood of signs and symptoms being recorded in the structured EHR data, (2) evidence of a diagnostic odyssey and therefore potential for impact by accelerating diagnosis, (3) opportunities for timely intervention if diagnosed early, (4) published diagnostic criteria or clinical suspicion criteria, among others.

42 case-finding algorithms were developed for 34 distinct rare diseases; for some diseases more than one algorithm was generated, to consider different clinical presentations or to comprise different methods of targeting the same disease.

Case-finding algorithms were developed using a combination of existing diagnostic or suspicion criteria and the input of disease experts. Disease features were selected and characterised, including collating comprehensive lists of appropriate SNOMED codes, defining the necessary logic such as age-specific onset or frequency of recurrence, and considering feature-specific exclusions. Clinical insights were combined with insights from available data, such as frequency of use of certain clinical codes. If certain codes or features were found to be infrequently recorded in EHR data, suitable proxies were considered. Algorithm structure and logic was formulated based on the characteristics of the disease and its features, and included simpler rules-based algorithms and weighted scoring systems.

### Retrospective validation study

We developed a case-control framework for assessing algorithm performance using the Optimum Patient Care Research Database (OPCRD) [19]. OPCRD holds de-identified, primary care EHR data from over 1,100 practices in the United Kingdom, with 24 million patients at the time of analysis. Permission for this study was granted by OPCRD’s Anonymised Data Ethics and Protocol Transparency Committee (ADEPT) (reference 1221).

### Data extraction and processing

Diagnostic codes (SNOMED codes and equivalent CTV3) codes were selected for the diseases of interest. A full list of all diagnostic codes used is provided in Additional File 1.

For each algorithm, cases were defined by the presence of a diagnostic SNOMED or CTV3 code, and all cases present in OPCRD were used. Though for some diseases few patients were present, all algorithms were still run on the relevant extract in order to represent the full spectrum of disease prevalence. Their diagnostic date was calculated as the date a diagnostic code first appeared in the EHR.

The control group consisted of two million patients selected randomly from OPCRD. Each case-control analysis was performed using the entire control group, removing only those controls who also were cases for the disease of interest.

A flagged patient was defined as output from an algorithm suggesting that a specific patient should be reviewed for the disease of interest.

Data was extracted using Structured Query Language (SQL) to retrieve essential patient details (patient ID, practice ID, year of birth, sex), and all clinical events for the patients of interest. All CTV3 codes in the patient records were mapped to the relevant SNOMED code, and all further analysis was carried out using the SNOMED codes only. Mapping files for CTV3 to SNOMED codes were generated from SNOMED CT UK Edition reference files [20]. As only birth year is available in OPCRD, patients were assigned a birth date of 2nd July of the year of birth.

### Key performance metrics

The following performance metrics were captured: sensitivity, specificity, flag rate, positive and negative likelihood ratios (LR+ and LR-), and positive predictive value (PPV).

Two methods of calculating the PPV were used: a prevalence-adjusted PPV utilising literature-based prevalence figures, and a prevalence-adjusted PPV using the prevalence of the disease in OPCRD (according to coded diagnosis). Unlike sensitivity and specificity, the PPV figure is influenced by the prevalence of the disease in the study population. The lower the disease prevalence in the population being tested, the more likely the algorithm is to encounter a patient without the disease, the greater the chance of a false positive and the lower the PPV. In case-control studies like this one, where the case / control ratio does not reflect the general population, the PPV must therefore be adjusted using the disease prevalence to calculate its expected value when the algorithm is deployed in the general population.

Prevalence figures were derived from published literature, prioritising UK data where possible; sources are detailed in Additional File 2. The prevalence of disease in the OPCRD (according to coded diagnosis) was also calculated. Adjusted PPV values using both prevalence figures were calculated.

In addition to the above key metrics, we also sought to undertake a time-based analysis. The earliest point at which each case would have been flagged was determined, allowing the calculation of the proportion of cases flagged before diagnosis, and the median time to be calculated.

## Results

### Sample summary

There were 2,036,489 individuals in this study: 39,655 cases (i.e. patients with one of the 34 diseases) and 2,000,000 controls, with some overlap where patients with a relevant diagnosis acted as a case for the relevant disease, and a control for the others. Across all individuals there were 870,891,352 total clinical event records.

### Algorithm performance

As expected, performance varies from metric to metric for the different algorithms and conditions. The full results for all 42 algorithms are presented in Additional File 3.

### Sensitivity

**Fig. 1:**
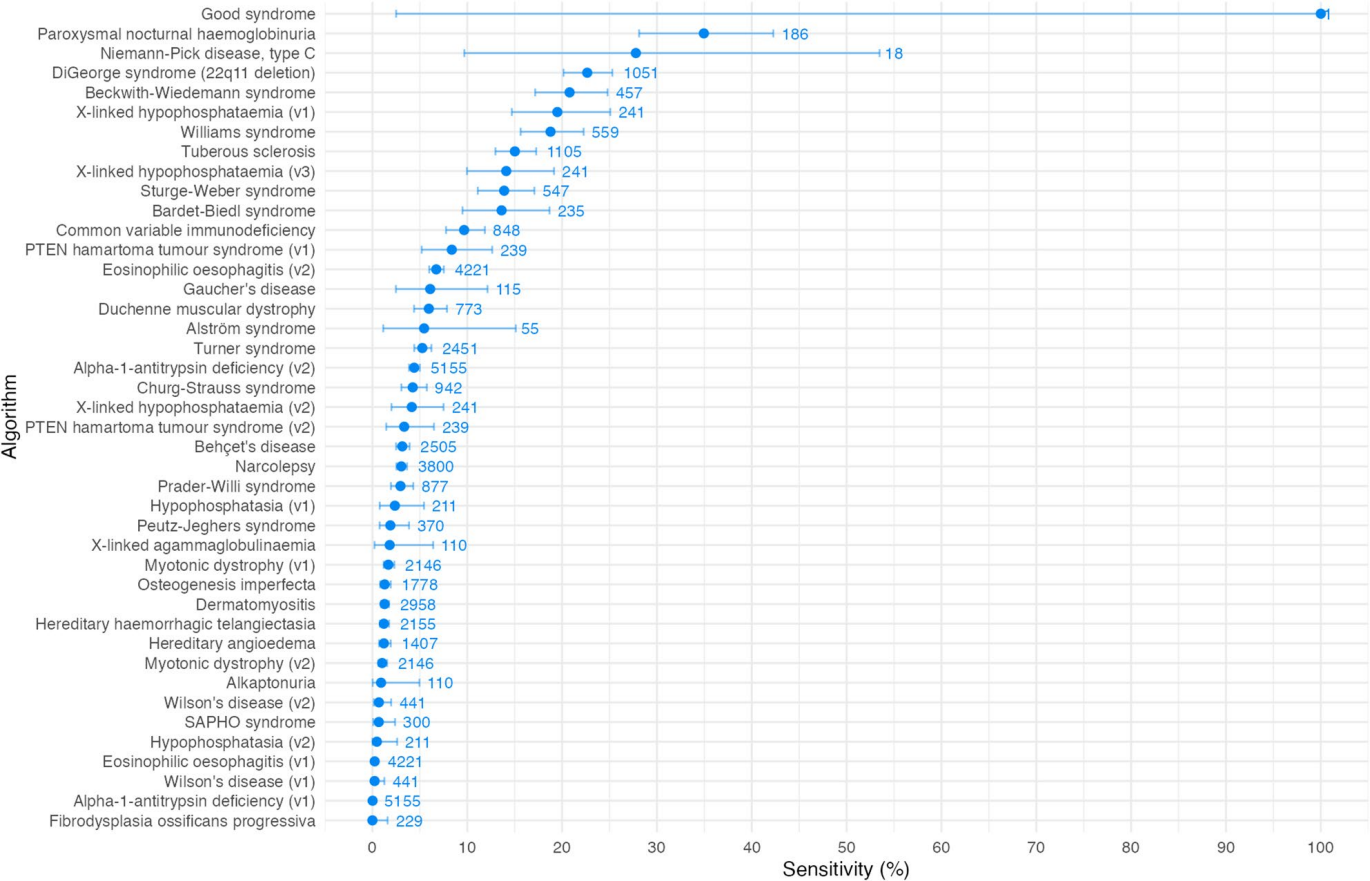
Algorithm performance by sensitivity i.e. the proportion of cases correctly flagged by each algorithm. Error bars represent 95% confidence intervals. Values on plot represent number of cases analysed. Where multiple versions of an algorithm were tested, algorithms are shown alongside their version number

Algorithm sensitivity, the proportion of known cases identified by each algorithm, is shown in Fig. 1. As can be seen in the figure, sensitivity ranged from 0 to 100%, but the majority were under 25%. The median sensitivity was 3.8% (IQR: 1.2–12.6%).

### Specificity

**Fig. 2:**
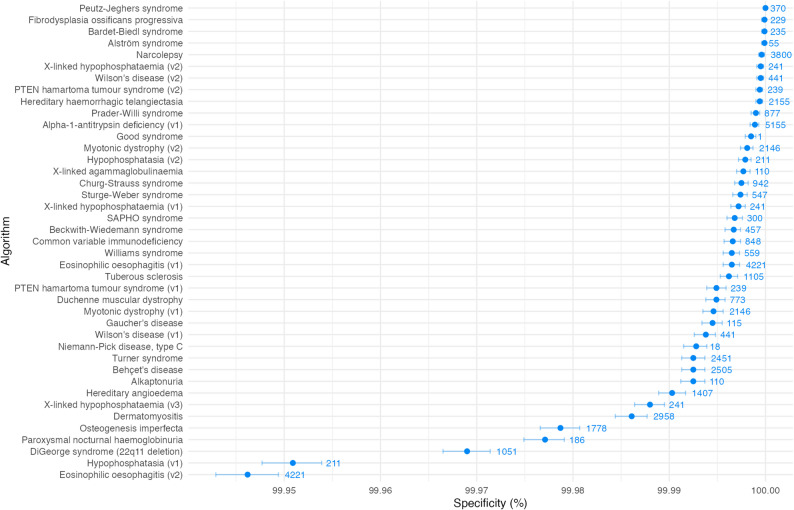
Algorithm performance by specificity i.e. the proportion of controls correctly not flagged by the algorithm. Error bars represent 95% confidence intervals. Values on plot represent number of cases analysed. Where multiple versions of an algorithm were tested, algorithms are shown alongside their version number

Results on the specificity metric, i.e. the proportion of controls correctly not flagged by the algorithm, are shown in Fig. 2. Most algorithms perform very well in this category, with a specificity above 99.995%. The median specificity was 99.9966% (IQR: 99.9925–99.9988). Alpha-1 antitrypsin deficiency (v2) flags a greater number of controls and has a lower specificity of 99.32%. This algorithm is not shown in Fig. 2 to allow a useful scale; a version of the plot with this included is present in Additional File 4.

### Positive predictive value (PPV)

**Fig. 3:**
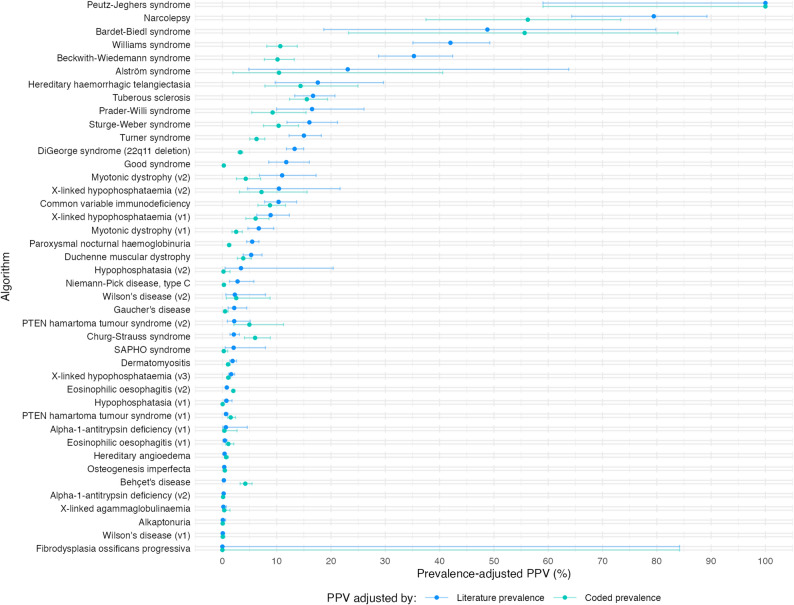
Algorithm performance by Positive Predictive Value (PPV), i.e. the proportion of flagged patients who are true cases. Error bars represent 95% confidence intervals

Algorithm performance as measured by PPV, i.e. the proportion of flagged patients who are true cases, adjusted by both literature prevalence and coded prevalence to reflect the expected value in the general population, is shown in Fig. 3. The median PPV adjusted by literature prevalence was 3.1% (IQR: 0.7–14.6%). The median PPV adjusted by coded prevalence was 2.5% (IQR: 0.4–8.4).

### Flag rate

**Fig. 4:**
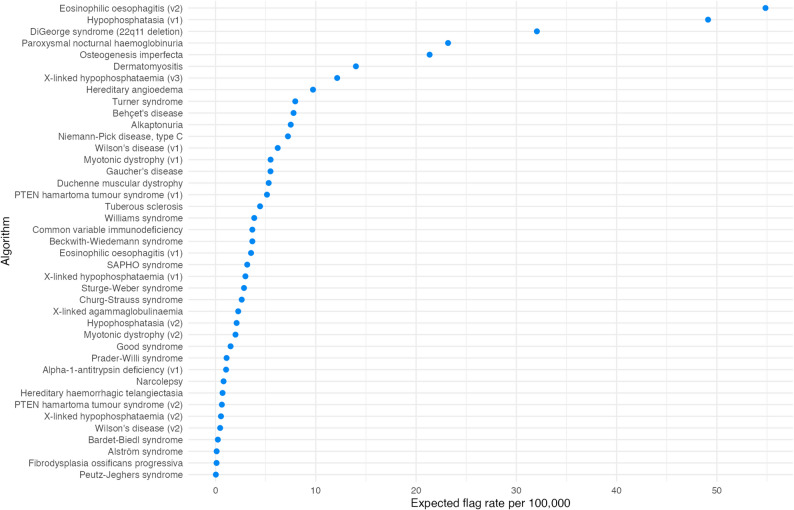
Algorithm performance by expected flag rate per 100,000. The flag rate is the number of patients expected to be flagged for review by the algorithm

Figure 4 shows the expected flag rate per 100,000 patients. Flag rates varied from 0.03 to 679 per 100,000, but the majority were under 20 per 100,000. The median flag rate was 3.7 per 100,000 (IQR: 1.2–7.7). One algorithm, Alpha-1-antitrypsin deficiency v2, had a flag rate of 679 per 100,000 and is not shown in Fig. 4. A version of the plot with this included is present in Additional File 4.

### Likelihood ratios

40 of the 42 algorithms had a positive likelihood ratio (LR+) above 10, and 34 of 42 had a value above 100. LR+ values had a median of 1167 (IQR: 125–4006), indicating that a positive result gives a strong signal for disease presence. All algorithms’ negative likelihood ratios (LR-) were close to 1, with a median of 0.96 (IQR: 0.87–0.99), reflecting limited clinical utility of a negative result. The LR + and LR- values are shown in Additional File 3.

### Temporal analysis

**Fig. 5:**
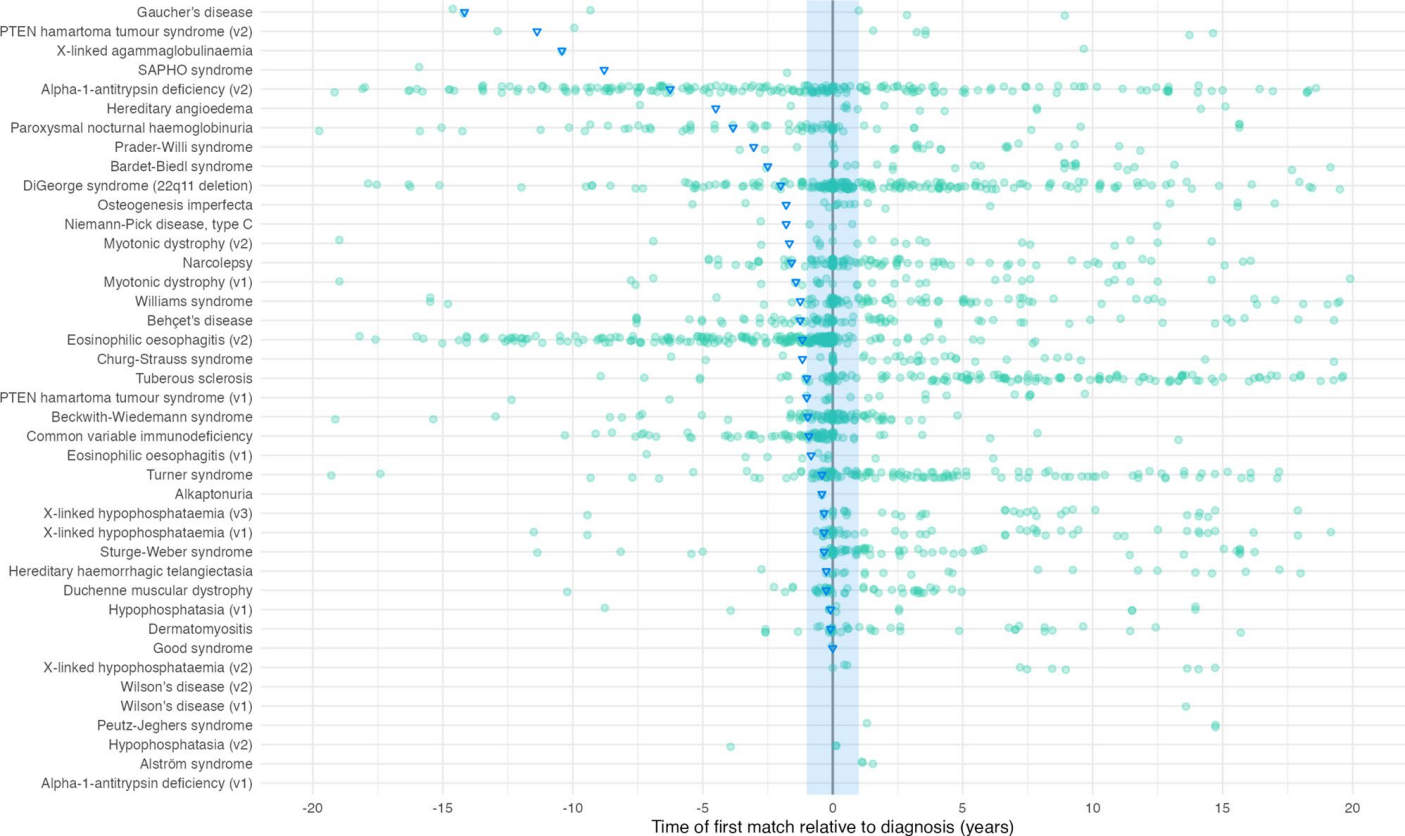
Time that the algorithms would have first flagged patients relative to their coded diagnosis. Each dot represents a patient. The blue shaded section represents 1 year before and after the coded diagnosis. Triangles represent the median relative time of patient flagging, of only the patients flagged *before* their diagnosis only. Plot is zoomed to focus on only the 20 years before and after diagnosis, but medians have been calculated using all points. 6% of total points fall outside this window

Fig. 5 shows that time of flagging relative to diagnosis is variable between algorithms. For some algorithms, such as eosinophilic oesophagitis (v2), most cases were flagged before diagnosis date, whereas for some, such as Turner syndrome, most were flagged after. Of the patients flagged before diagnostic date across all diseases, the median time flagged ranged from 1 to 170 months before diagnosis

## Discussion

In this retrospective validation study, we assessed the analytical validity of 42 case finding algorithms for 34 rare diseases, to understand their potential application in the real world to identify patients with undiagnosed rare diseases.

Here, we discuss further each performance metric.

### Sensitivity

We expected sensitivity to be limited, as structured EHR data is often sparse, and some patients’ structured records have very few detectable clinical features present, limiting the number of cases who are findable through their structured EHR data.

Sensitivity ranged from 0 to 100% across the 42 algorithms, with the majority under 25%. Sensitivity is higher in diseases where certain characteristic features are both typically present and captured in a structured format in the EHR.

The algorithm for DiGeorge syndrome, a microdeletion syndrome with heterogeneous presentation for which diagnostic delays are common [21, 22], is one of the top-performing algorithms for sensitivity, with a value of 22.65% (CI 20.15–25.30%). This is likely to be driven by the fact that many of the features of interest, such as cardiac conditions, palate abnormalities and immunodeficiencies, are likely to be coded in the structured EHR data and therefore flagged by the algorithm.

Where algorithms rely on features that are not well recorded in a structured format in the EHR, sensitivity can be poor. An example is the algorithm for hypophosphatasia (v2), which incorporates blood test results such as phosphate, calcium, and alkaline phosphatase. The algorithm focuses on diagnosis of paediatric hypophosphatasia and hence focuses on features below the age of 16, where these blood test results are both not frequently performed and if they have been, performed in primary care and therefore found in the primary care EHR. Algorithm sensitivity is therefore low.

Maximising algorithm sensitivity often comes at the cost of reducing specificity and PPV to a level where algorithm deployment would not be feasible. Our aim is not necessarily to flag *all* undiagnosed patients, but to identify as many patients for review as it is feasible to find using their structured EHR data, while maintaining a PPV suitable for scalable deployment. Despite such algorithms not being designed to detect all undiagnosed patients, they are still expected to bring clinical utility with low resource requirements to the healthcare systems.

### Specificity

In contrast to sensitivity, high specificity is necessary for an algorithm to be viable for deployment at scale. For rare diseases, the far greater number of controls than cases in the general population means that even if a tiny proportion of controls are flagged, the number flagged may still be unfeasibly high for clinician review. Where the prevalence of the cases in the general population is low, as with rare diseases, specificity must be close to 100% for the resulting PPV to be acceptably high. Algorithms therefore are designed to perform with high specificity, for example, to require clusters of features that rarely occur in patients without the disease. Our results showed that most algorithms therefore perform well in this category, with the majority above 99.995%.

### PPV

PPV is probably the most important metric, as it indicates the viability of deploying the algorithm in primary care. A reasonable proportion of patients flagged should be patients with the disease, in order for clinical review of flagged patients to be a reasonable use of time and resource.

Some algorithms perform with a high PPV; one example is Williams syndrome, a genetic disorder characterised by distinctive facial features, cardiovascular disease, developmental delays, and a unique cognitive profile with strong language skills and an overly friendly personality [23]. The Williams syndrome algorithm has the best PPV of 10.67% (8.18–13.79) when adjusted for coded prevalence and 42.00% (35.09–49.24) when adjusted for literature prevalence.

The small numbers of cases for some diseases, such as Peutz-Jeghers syndrome and fibrodysplasia ossificans progressiva, leaves uncertainty in the estimated PPV. However, both algorithms may still offer clinical utility: even if a small number of the screened population are flagged (therefore requiring limited resources to review), a PPV of even 10%, for example, would represent that 1 in 10 of any flagged cases is a true undiagnosed patient, which may represent a reasonable investment of resource for what are difficult and hard to diagnose rare diseases.

As can be seen on the plot, there can be large differences between the PPV values for a particular algorithm when adjusted for literature-based prevalence or coded prevalence. One example is Beckwith-Wiedemann syndrome, with a PPV of 35.3% (literature-based) or 10.2% (coded). As with many of the diseases, it is possible that many true patients in the study dataset are either undiagnosed, or have a diagnosis that has not been coded in their EHR. The coded prevalence, and hence the coded PPV value, is therefore likely to be an underestimate. It is important to note that even the best estimates of rare disease prevalence available in the literature often have significant uncertainty.

### Flag rate

Flag rate is an important metric when it comes to evaluating the potential feasibility of deploying case-finding tools in real-world settings. A high flag rate indicates that an algorithm may be impractical for deployment at scale, due to the resources it would require to review all flagged patients. Flag rates varied from 0.03 to 679 per 100,000, but the majority were under 20 per 100,000. Flag rates of this order are more likely to be suitable for deployment in primary care settings. Those with significantly higher flag rates would not be considered suitable for deployment in their current state.

### Positive and negative likelihood ratios

To better characterise the strength of the algorithms to surface patients of interest, independent of disease prevalence, we calculated the Positive Likelihood Ratio (LR+), which indicates how much more likely a positive flag is among patients with the disease compared to those without it, and the Negative Likelihood Ratio (LR−), which indicates the utility of a negative flag in ruling out the disease.

Many of the algorithms showed a highly significant clinical impact for positive results, with a median LR + of 1167 (IQR: 125–4006). 40 of 42 performed with an LR+ above 10, and 34 with an LR+ above 100. For these algorithms, a flagged patient is over a hundred times more likely to have the disease of interest, justifying these patients being prioritised for specialist review. This high LR + is driven by the algorithms’ very high specificity.

Conversely, with LR- values all close to 1 (median 0.96), the algorithms provide very limited ability to “rule out” a disease: a negative result provides almost no evidence that a patient does not have the rare disease, and hence should not be used to exclude a diagnosis. This is a consequence of the algorithms’ relatively low sensitivity. This meets the intended use of these algorithms: their purpose is not to diagnose patients nor to rule out disease, but to act as filters that maximise the probability of identifying true cases for review and potential testing, and hence increasing the chance of earlier diagnosis.

### Temporal analysis

There is significant variability in the time of flagging relative to diagnosis between algorithms, as shown in Fig. 5. Of the patients flagged before diagnosis, the median time ranged from 1 to 170 months. However, it is important to note that the median flag time needs to be considered in context of other metrics. The Gaucher’s disease algorithm had a before diagnosis median time of 170 months, but this was based on only three patients (43% of the flagged patients) and other metrics for this algorithm were less impressive: PPV values were 2.2% and 0.5% (adjusted for literature prevalence and coded prevalence respectively).

Conversely, the algorithm for common variable immunodeficiency (CVID) performs well in terms of time-based and other metrics. 81.7% of patients in the validation dataset were flagged prior to coded diagnosis, 39% flagged 1 year or more prior to diagnosis, and the median time flagged of those flagged before diagnosis was 11 months. The algorithm performs with a sensitivity of 9.7% and PPV values of 10.4% and 8.8% (adjusted for literature prevalence and coded prevalence respectively).

### Significance of findings for future research and deployment

This retrospective study is the first step in evaluating the utility of this case-finding approach to improve and expedite diagnosis of rare diseases. Further research is now ongoing to deploy the tool in real-world settings and to obtain clinician and patient feedback on its utility. This approach is guided by independent standards on developing an evidence base for digital tools in healthcare, including the NICE Evidence Standards Framework [19]. Thus, although much learning has been gained from this exercise and many of the findings appear to show promise, additional structured work needs to be undertaken to evaluate this approach, which will then inform decisions on widespread use. Therefore, any conclusions drawn from this work are tentative, and subject to refinement as the evidence continues to be built from multiple sources.

Below, we discuss the merits and limitations of our approach, the significance of these findings and their implications for future research and clinical deployment.

#### Are these results representative of how this approach might work in clinical settings?

This large retrospective study uses a dataset of UK primary care EHR (2,036,489 patient EHRs), so gives real insights into how these algorithms may work in real-world settings. However, like any EHR data study there are recognised limitations.

For instance, we make reasonable assumptions about diagnostic coding, i.e. that those with a diagnostic code in their structured EHR have a genuine diagnosis, and those without do not. However, some cases may be misclassified as controls. This may be due to a known case not having their diagnosis coded in their EHR, or as is common in rare disease, patients with the disease not having received a diagnosis. Similarly, some controls may have been misclassified as cases, due to diagnostic codes entered incorrectly. These limitations of ascertainment are acknowledged and highlight the importance of capturing diagnostic outcomes for flagged cases in real world deployment. We have undertaken some pilot work in this area [24] and additional work to deploy these algorithms and evaluate them in clinical settings is underway.

There will always be difficulties associated with measuring algorithm performance as performance on each metric is typically related to performance on another. An algorithm’s PPV (proportion of flagged patients that are cases) is dependent on disease prevalence in the test population: a higher prevalence increases PPV as the algorithm is more likely to come across true patients. Our study was a case-control study where the proportion of cases present was enriched compared to the general population; due to this, calculating the unadjusted PPV would result in an over-estimate of the algorithm’s true PPV; i.e. its value if deployed in the general population. To adjust for this, we calculated prevalence-adjusted PPV figures [25]. However, this also comes with challenges, as true prevalence is often not known with much precision, particularly for very rare diseases.

All of these factors affect how we evaluate algorithm performance and should be taken into consideration when reviewing the results.

#### What is good performance?

There is no absolute threshold that indicates whether an algorithm performs “well” or “badly”, and interpreting what could be considered as good performance is contextual. There are also nuances involved in assessing public health interventions, such as trade-offs between sensitivity vs. specificity, and constraints on clinical resources and budget. Due to this, we adopt a bespoke approach for each rare disease by considering the problem we are trying to solve and then evaluating performance through different lenses. Some specific examples are provided below.

One potentially informative metric is PPV. As a point of reference, an NHS standard for fast track cancer referrals uses clinical features with a PPV of 3% or greater. If evaluated using similar thresholds, many were high-performing: they performed with a high PPV and a high proportion of patients were found in advance of diagnosis. However, when it comes to rare diseases, using a cut-off criterion is not always advisable, particularly when confidence around the figure is limited. Our results show that fibrodysplasia ossificans progressiva has a PPV of 0%, as no cases were flagged. Although the algorithm was run on 229 “cases”, the diagnostic code used for this disease is non-specific, also incorporating other types of progressive myositis ossificans, and prevalence figures from literature suggest fewer than 20 true cases within this dataset. This observed PPV of zero may therefore be influenced by the limited number of patients who truly have FOP. While no cases were flagged here, the algorithm was designed based on robust evidence of physical features in FOP cases, and deployment in a large population may result in the identification of some positive cases. As early diagnosis of FOP is crucial for good prognosis, we believe that there is a rationale to deploy it in real-world settings, subject to a more complete real world evaluation.

Another consideration is the sensitivity vs. specificity trade-off. High specificity scores for most algorithms is a promising indicator that we can effectively sift out cases that are not relevant, and are unlikely to overburden services when deployed. Lower sensitivity scores are to be expected, as these are dependent on detectable clinical features present in often-sparse EHR data.

As mentioned previously, how we evaluate performance varies from disease to disease and the problem at hand. For example, in DiGeorge syndrome, we have developed the algorithm to focus on patients *without* the subtype comprising more florid congenital malformations, who are more likely to have been heavily investigated and therefore a lower need for case-finding. Although the algorithm could be built to increase sensitivity by focusing on this subtype as well, this could sacrifice specificity, making it impractical to implement. Moreover, we would not be working toward solving the problem of detecting the cohort of patients who do not get diagnosed in a timely manner. This is an apt example demonstrating that each metric is best evaluated on a disease-by-disease basis.

Our time-based analysis shows that a large proportion of patients are first flagged after their coded diagnosis. This is expected as for many patients, information on symptoms and tests are only added to their record after a confirmed diagnosis. However, our results demonstrate that many of our algorithms can also detect patients *prior* to diagnosis. This is a significant finding as it confirms that specific phenotypes that are missed can be detected in advance.

In summary, we propose that instead of using performance metrics in isolation, optimal decisions should be made for each disease taking into account evidence from across the varying performance metrics in addition to knowledge of the clinical pathway and findings from real world evaluations, as suggested by the NICE Evidence Standards Framework [19].

#### How can the performance of EHR case-finding algorithms be improved?

The algorithms tested are subject to continuous optimisation to improve performance. Regular review of the literature will ensure that emerging codes and disease features are incorporated into the algorithms.

Future improvements in structured EHR data capture could contribute to improved algorithm performance. This could include: (1) improvement in clinical awareness of how to accurately and systematically code data, (2) consolidation of structured EHR data across primary and secondary care systems, enabling confirmation of diagnosis in other datasets, (3) integration and transformation of unstructured EHR data into a structured format.

The outcomes from validation rely heavily on the structured EHR data being available, accurate and accessible. Some diseases are ultra-rare and there is not enough data available in public databases to use with such algorithms. For instance, there was only 1 case of Good syndrome in the OPCRD record.

Our time-based analysis relies on the assumption that the date on the EHR is a good proxy of when patients received their diagnosis. Choice of diagnostic codes is also important. Broader codes that encompass the disease of interest are sometimes available, but it is best to use only codes specific to the disease to ensure representative performance metrics. Advances in statistics and machine learning, as well as policy changes making EHR data more accessible, offer the possibility of improving these algorithms further. Outcomes from our real-world evaluation work will also inform future algorithm development.

#### What else do we need to consider when interpreting these findings?

A few other factors should be taken into consideration when reviewing these findings. Our algorithms have been designed to identify patients with undiagnosed rare diseases. But due to the nature of the study dataset, these algorithms can only be tested for their performance in identifying patients with diagnosed rare diseases. The EHR data for both these groups of patients, i.e. those with a confirmed rare disease diagnosis vs. those that are yet to be diagnosed, will be different. For example, those with a diagnosis may be more likely to have features of interest coded in their EHR, especially after their diagnosis is known. This limitation is mitigated but not eliminated by assessing which patients would have been flagged before their coded diagnosis, and how long beforehand.

#### What are the next steps?

There is much potential for further work in this area. The field is constantly evolving and new developments in the use of AI and machine learning, in addition to advances in EHR data coding and availability, will allow for the development of new and improved algorithms. A key factor that could impact real-world translation is identifying which diseases are prime candidates that could benefit from such algorithms. This requires collaboration with key opinion leaders for each disease and awareness of specific patient pathways. For instance, for certain diseases, there are pieces of information available in the EHR which make them good candidates for use with diagnostic tools. Findings from our other work streams e.g. real-world deployment and patient and clinician acceptability will add to our findings from this analytical validation. Larger real world evaluations are required in order to fully ascertain the impact of such case-finding algorithms in assisting with the detection and diagnosis of patients with rare diseases.

## Supplementary Information

Below is the link to the electronic supplementary material.

Supplementary Material 1

Supplementary Material 2

Supplementary Material 3

Supplementary Material 4

## Data Availability

Due to data access licensing and data security, raw study data cannot be made available to other investigators. Regulations regarding access to and use of the Optimum Patient Care Research Database are available here: https://www.opcrd.optimumpatientcare.org/licenses.
