# Supplementary material for: Evaluating algorithmic approaches to rare disease case-finding: a retrospective validation study using electronic health records
Source: Orphanet J Rare Dis. 2026 Feb 4;21:120. doi: 10.1186/s13023-026-04240-6 (PMC13041464; doi:10.1186/s13023-026-04240-6)
Supplement: Supplementary file 1 — Supplementary Material 1 [file 13023_2026_4240_MOESM1_ESM.pdf]

Additional file 1

Diagnostic codes used to define validation case groups

Active status and date of last status change are here available only for SNOMED codes.
For active codes, the date of last status change is the date the code was introduced; for inactive codes it is the date it was made inactive.
This table shows only the preferred term for SNOMED codes and not all synonyms. Diagnostic codes may have been chosen due to one of the synonyms rather than the preferred term.

Disease	Ontology	Concept ID	Preferred term	Active Status	Date of last status change
Alkaptonuria	SNOMED	360378009	Homogentisate 1,2-dioxygenase deficiency	Active	2002-01-31
Alkaptonuria	SNOMED	410042009	Alcaptonuric ochronosis	Active	2004-07-31
Alkaptonuria	SNOMED	24250001	Alkaptonuria	Inactive	2002-01-31
Alkaptonuria	SNOMED	190689001	Homogentisic acid defect (& alkaptonuria)	Inactive	2002-01-31
Alkaptonuria	SNOMED	403263009	Ochronosis due to alkaptonuria	Inactive	2004-07-31
Alkaptonuria	SNOMED	360381004	Alkaptonuria	Inactive	2020-07-31
Alkaptonuria	CTV3	C3020	Homogentisic acid defect (&++ | Homogentisic acid defect (& alkaptonuria)	-	-
Alkaptonuria	CTV3	X40Rk	Homogentisic acid defect	-	-
Alkaptonuria	CTV3	XE116	Homogentisate 1,2-dioxygen def | Homogentisate 1,2-dioxygenase deficiency	-	-
Alpha-1-antitrypsin deficiency	SNOMED	30188007	Alpha-1-antitrypsin deficiency	Active	2002-01-31
Alpha-1-antitrypsin deficiency	SNOMED	154771007	Alpha-1-antitrypsin deficiency	Inactive	2002-01-31
Alström syndrome	SNOMED	63702009	Alstrom syndrome	Active	2002-01-31
Alström syndrome	SNOMED	112841000000102	Alstrom syndrome	Inactive	2004-07-31
Alström syndrome	CTV3	Xa0Zf	Alstrom syndrome	-	-
Bardet-Biedl syndrome	SNOMED	5619004	Bardet-Biedl syndrome	Active	2002-01-31
Bardet-Biedl syndrome	CTV3	PKy1.	Laurence-Moon-Biedl syndrome	-	-
Beckwith-Wiedemann syndrome	SNOMED	81780002	Beckwith-Wiedemann syndrome	Active	2002-01-31
Beckwith-Wiedemann syndrome	CTV3	PKy91	Beckwith's syndrome	-	-
Behçet's disease	SNOMED	3275009	Behcet's syndrome, vascular type	Active	2002-01-31
Behçet's disease	SNOMED	21542005	Behcet's syndrome, neurologic type	Active	2002-01-31
Behçet's disease	SNOMED	53485006	Behcet's syndrome, incomplete type	Active	2002-01-31
Behçet's disease	SNOMED	57484009	Behcet's syndrome, complete type	Active	2002-01-31
Behçet's disease	SNOMED	201485008	Arthropathy in Behcet's syndrome of the shoulder region	Active	2002-01-31
Behçet's disease	SNOMED	201488005	Arthropathy in Behcet's syndrome of the hand	Active	2002-01-31
Behçet's disease	SNOMED	201489002	Arthropathy in Behcet's syndrome of the pelvic region and thigh	Active	2002-01-31
Behçet's disease	SNOMED	201491005	Arthropathy in Behcet's syndrome of the ankle and/or foot	Active	2002-01-31
Behçet's disease	SNOMED	201492003	Arthropathy in Behcet's syndrome of multiple sites	Active	2002-01-31
Behçet's disease	SNOMED	235758007	Behcet's colitis	Active	2002-01-31
Behçet's disease	SNOMED	239921005	Behcet's disease with organ/system involvement	Active	2002-01-31
Behçet's disease	SNOMED	239922003	Behcet's disease with multisystem involvement	Active	2002-01-31
Behçet's disease	SNOMED	310701003	Behcet's disease	Active	2002-01-31
Behçet's disease	SNOMED	410480004	Iritis in Behcet's syndrome	Active	2004-07-31
Behçet's disease	SNOMED	410481000	Panuveitis in Behcet's syndrome	Active	2004-07-31
Behçet's disease	SNOMED	62918002	Arthropathy in Behcet's syndrome	Active	2005-01-31
Behçet's disease	SNOMED	198231000	Ulceration of vulva in Behcet's disease	Active	2006-01-31
Behçet's disease	SNOMED	198231000	Ulceration of vulva in Behcet's disease	Active	2006-01-31
Behçet's disease	SNOMED	427967009	Arthropathy in Behcet's syndrome of the spine	Active	2008-01-31
Behçet's disease	SNOMED	428169009	Arthropathy in Behcet's syndrome of the ankle	Active	2008-01-31
Behçet's disease	SNOMED	54034003	Behcet's syndrome, intestinal type	Active	2020-07-31
Behçet's disease	SNOMED	403443000	Behçet's disease affecting oral mucosa	Active	2020-07-31
Behçet's disease	SNOMED	866049001	Mucocutaneous Behçet disease	Active	2020-07-31
Behçet's disease	SNOMED	1144914004	Behcet disease of skin	Active	2021-07-31
Behçet's disease	SNOMED	1144919009	Behcet disease of eye	Active	2021-07-31
Behçet's disease	SNOMED	1144959007	Ulceration of scrotum due to Behcet disease	Active	2021-07-31
Behçet's disease	SNOMED	1144971001	Ulcer of small intestine due to Behcet syndrome	Active	2021-07-31
Behçet's disease	SNOMED	1144983004	Anogenital ulceration due to Behcet disease	Active	2021-07-31
Behçet's disease	SNOMED	1142107003	Transient neonatal Behçet disease	Active	2021-07-31
Behçet's disease	SNOMED	1144983004	Anogenital ulceration due to Behcet disease	Active	2021-07-31
Behçet's disease	SNOMED	403472004	Penile ulceration due to Behçet's disease	Active	2021-10-31
Behçet's disease	SNOMED	403472004	Penile ulceration due to Behçet's disease	Active	2021-10-31
Behçet's disease	SNOMED	724783000	Demyelination of central nervous system due to Behcet disease	Active	2023-03-31
Behçet's disease	SNOMED	154424000	Behcet's disease	Inactive	2002-01-31
Behçet's disease	SNOMED	201484007	Arthropathy in Behcet's syndrome (& [unspecified site])	Inactive	2002-01-31
Behçet's disease	SNOMED	41225007	Behçet's syndrome	Inactive	2002-01-31

Disease	Ontology	Concept ID	Preferred term	Active Status	Date of last status change
Behçet's disease	SNOMED	201493008	Arthropathy in Behcet's syndrome of other specified sites	Inactive	2010-01-31
Behçet's disease	SNOMED	201494002	Arthropathy in Behcet's syndrome NOS	Inactive	2010-01-31
Behçet's disease	SNOMED	267884002	Arthropathy in Behcet's syndrome of unspecified site	Inactive	2010-01-31
Behçet's disease	SNOMED	594541000000104	Arthropathy in Behcet's syndrome of other specified sites	Inactive	2011-04-01
Behçet's disease	SNOMED	598241000000107	Arthropathy in Behcet's syndrome NOS	Inactive	2012-04-01
Behçet's disease	SNOMED	201486009	Arthropathy in Behcet's syndrome of the upper arm	Inactive	2012-07-31
Behçet's disease	SNOMED	201487000	Arthropathy in Behcet's syndrome of the forearm	Inactive	2012-07-31
Behçet's disease	SNOMED	201490006	Arthropathy in Behcet's syndrome of the lower leg	Inactive	2012-07-31
Behçet's disease	SNOMED	567011000000103	Arthropathy in Behcet's syndrome of unspecified site	Inactive	2012-10-01
Behçet's disease	SNOMED	403481005	Vulval ulceration due to Behçet's disease	Inactive	2015-07-31
Behçet's disease	SNOMED	1144958004	Ulceration of penis due to Behcet disease	Inactive	2021-10-31
Behçet's disease	CTV3	N012.	Arthrop+Behcet's syndrome | Arthropathy in Behcet's syndrome	-	-
Behçet's disease	CTV3	N0120	Arthropathy in Behcet's synd++ | Arthropathy in Behcet's syndrome (& [unspecified site])	-	-
Behçet's disease	CTV3	N0123	Arthrop+Behcet's synd-forearm | Arthropathy in Behcet's syndrome of the forearm	-	-
Behçet's disease	CTV3	N0126	Arthrop+Behcet's synd-leg | Arthropathy in Behcet's syndrome of the lower leg	-	-
Behçet's disease	CTV3	N012y	Arthrop+Behcet's synd-oth spec | Arthropathy in Behcet's syndrome of other specified sites	-	-
Behçet's disease	CTV3	N012z	Arthrop+Behcet's synd NOS | Arthropathy in Behcet's syndrome NOS	-	-
Behçet's disease	CTV3	XE1DQ	Arthrop+Behcet's synd-unspec | Arthropathy in Behcet's syndrome of unspecified site	-	-
Behçet's disease	CTV3	K4252	Ulceratn vulva/Behcet's diseas | Ulceration of vulva in Behcet's disease	-	-
Behçet's disease	CTV3	N0121	Arthrop+Behcet's synd-shoulder | Arthropathy in Behcet's syndrome of the shoulder region	-	-
Behçet's disease	CTV3	N0122	Arthrop+Behcet's synd-upp arm | Arthropathy in Behcet's syndrome of the upper arm	-	-
Behçet's disease	CTV3	N0124	Arthrop+Behcet's synd-hand | Arthropathy in Behcet's syndrome of the hand	-	-
Behçet's disease	CTV3	N0125	Arthrop+Behcet's synd-pelv/thi | Arthropathy in Behcet's syndrome of the pelvis/thigh | Arthropathy in Behcet's syndrome of the pelvic region and thigh	-	-
Behçet's disease	CTV3	N0127	Arthrop+Behcet's synd-ank/foot | Arthropathy in Behcet's syndrome of the ankle and foot	-	-
Behçet's disease	CTV3	N012x	Arthrop+Behcet's synd-multiple | Arthropathy in Behcet's syndrome of multiple sites	-	-
Behçet's disease	CTV3	X3046	Behcet's colitis	-	-
Behçet's disease	CTV3	X705J	Behcet dis w organ/system invl | Behcet's disease with organ/system involvement	-	-
Behçet's disease	CTV3	X705K	Behcet dis w multisyst involv | Behcet's disease with multisystem involvement	-	-
Behçet's disease	CTV3	AD61.	Behcet's disease	-	-
Common variable immunodeficiency	SNOMED	191013002	Common variable immunodeficiency with autoantibodies to B- or T-cells	Active	2002-01-31
Common variable immunodeficiency	SNOMED	191012007	Common variable immunodeficiency with predominant immunoregulatory T-cell disorders	Active	2002-01-31
Common variable immunodeficiency	SNOMED	191011000	Common variable immunodeficiency with predominant abnormalities of B-cell numbers and functions	Active	2002-01-31
Common variable immunodeficiency	SNOMED	23238000	Common variable agammaglobulinaemia	Active	2002-01-31
Common variable immunodeficiency	SNOMED	773646003	PLCG2-associated antibody deficiency and immune dysregulation	Active	2019-01-31
Common variable immunodeficiency	SNOMED	191010004	Common variable immunodeficiency	Active	2023-07-31
Common variable immunodeficiency	SNOMED	191031001	[X]Other common variable immunodeficiencies	Inactive	2009-01-31
Common variable immunodeficiency	SNOMED	411431000000100	[X]Other common variable immunodeficiencies	Inactive	2015-04-01
Common variable immunodeficiency	CTV3	C3982	Cm vr imdef autantbod B/T-cell | Common variable immunodef wth autoantibod to B- or T-cells | Common variable immunodeficiency with autoantibodies to B- or T-cells	-	-
Common variable immunodeficiency	CTV3	C3981	Cm vr imdef prdm imreg T-disrd | Common var immunodef predom immunoregulatory T-cell disorder | Common variable immunodeficiency with predominant immunoregulatory T-cell disorders	-	-
Common variable immunodeficiency	CTV3	C3980	Cm vr imdef prdm abn B-c nos+f | Com var immunodef with predom abn B-cell numbers and functns | Common variable immunodeficiency with predominant abnormalities of B-cell numbers and functions	-	-
Common variable immunodeficiency	CTV3	C3907	Common variable immunodefic | Common variable immunodeficiency	-	-
Dermatomyositis	SNOMED	238935002	Dermatomyositis sine myositis	Active	2002-01-31
Dermatomyositis	SNOMED	238936001	Sclerodermatomyositis	Active	2002-01-31
Dermatomyositis	SNOMED	396230008	Dermatomyositis	Active	2003-07-31
Dermatomyositis	SNOMED	239901009	Dermatomyositis with malignant disease	Active	2004-07-31
Dermatomyositis	SNOMED	46696008	Dilated cardiomyopathy due to dermatomyositis	Active	2010-07-31
Dermatomyositis	SNOMED	296241000119107	Disorder of respiratory system due to dermatomyositis	Active	2015-07-31
Dermatomyositis	SNOMED	402425006	Adult onset dermatomyositis	Active	2020-07-31
Dermatomyositis	SNOMED	838366009	Anti-synthetase syndrome due to dermatomyositis	Active	2020-07-31
Dermatomyositis	SNOMED	865923002	Assessment using Cutaneous Dermatomyositis Disease Area and Severity Index	Active	2020-07-31
Dermatomyositis	SNOMED	865924008	Cutaneous Dermatomyositis Disease Area and Severity Index	Active	2020-07-31
Dermatomyositis	SNOMED	1144911007	Calcinosis due to adult type dermatomyositis	Active	2021-07-31
Dermatomyositis	SNOMED	1144931006	Dermatomyositis overlap syndrome	Active	2021-07-31
Dermatomyositis	SNOMED	1153408000	Calcification of muscle due to adult dermatomyositis	Active	2021-07-31
Dermatomyositis	SNOMED	281358000	Idiopathic dermatomyositis	Active	2021-07-31

Disease	Ontology	Concept ID	Preferred term	Active Status	Date of last status change
Dermatomyositis	SNOMED	156456005	Dermatomyositis	Inactive	2002-01-31
Dermatomyositis	SNOMED	201445002	Dermatomyositis (& [Poikilodermatomyositis])	Inactive	2002-01-31
Dermatomyositis	SNOMED	201447005	Dermatomyositis with malignant disease	Inactive	2002-01-31
Dermatomyositis	SNOMED	38826005	Dermatomyositis	Inactive	2003-07-31
Dermatomyositis	SNOMED	203785004	[X]Other dermatomyositis	Inactive	2009-01-31
Dermatomyositis	SNOMED	396229003	Adult type dermatomyositis	Inactive	2015-01-31
Dermatomyositis	SNOMED	462101000000107	[X]Other dermatomyositis	Inactive	2015-04-01
Dermatomyositis	CTV3	N003.	Dermatomyositis (& [Poikilod++ | Dermatomyositis (& [Poikilodermatomyositis])	-	-
Dermatomyositis	CTV3	X50FJ	Dermatomyositis sine myositis	-	-
Dermatomyositis	CTV3	X50FL	Sclerodermatomyositis	-	-
Dermatomyositis	CTV3	Nyu48	[X]Dermat(poly)myosit/neo d CE | [X]Dermato(poly)myositis in neoplastic disease CE | [X]Dermato(poly)myositis in neoplastic disease classified elsewhere	-	-
Dermatomyositis	CTV3	X704t	Dermatomyos w malignant diseas | Dermatomyositis with malignant disease	-	-
Dermatomyositis	CTV3	Xa1jL	Idiopathic dermatomyositis	-	-
Dermatomyositis	CTV3	Nyu44	[X]Other dermatomyositis	-	-
Dermatomyositis	CTV3	Nyu4E	[X]Dermatopolymyositis, unspec | [X]Dermatopolymyositis, unspecified	-	-
Dermatomyositis	CTV3	XE1DH	Dermatomyositis	-	-
DiGeorge syndrome (22q11 deletion)	SNOMED	767263007	22q11.2 deletion syndrome	Active	2018-07-31
DiGeorge syndrome (22q11 deletion)	SNOMED	190991007	DiGeorge syndrome	Inactive	2002-01-31
DiGeorge syndrome (22q11 deletion)	SNOMED	77128003	DiGeorge sequence	Inactive	2018-07-31
DiGeorge syndrome (22q11 deletion)	SNOMED	460436001	22q11 microdeletion with complete DiGeorge sequence	Inactive	2018-07-31
DiGeorge syndrome (22q11 deletion)	SNOMED	449818005	22q11 deletion syndrome	Inactive	2021-09-30
DiGeorge syndrome (22q11 deletion)	CTV3	XaYQ0	Chromosome 22q11 deletion synd | Chromosome 22q11 deletion syndrome	-	-
DiGeorge syndrome (22q11 deletion)	CTV3	C3911	DiGeorge syndrome	-	-
DiGeorge syndrome (22q11 deletion)	CTV3	X00mm	Shprintzen syndrome	-	-
Duchenne muscular dystrophy	SNOMED	76670001	Duchenne muscular dystrophy	Active	2002-01-31
Duchenne muscular dystrophy	SNOMED	315608004	Cardiomyopathy in Duchenne muscular dystrophy	Active	2010-07-31
Duchenne muscular dystrophy	CTV3	F3910	Duchenne muscular dystrophy	-	-
Duchenne muscular dystrophy	CTV3	XaI9b	Cardiomyo Duchenne muscul dyst | Cardiomyopathy in Duchenne muscular dystrophy	-	-
Eosinophilic granulomatosis with polyangiitis (Churg-Strauss syndrome)	SNOMED	317931000119101	Pulmonary disease due to allergic granulomatosis angiitis	Active	2015-07-31
Eosinophilic granulomatosis with polyangiitis (Churg-Strauss syndrome)	SNOMED	82275008	Eosinophilic granulomatosis with polyangiitis	Active	2020-07-31
Eosinophilic granulomatosis with polyangiitis (Churg-Strauss syndrome)	SNOMED	195362002	Churg-Strauss vasculitis	Inactive	2002-01-31
Eosinophilic granulomatosis with polyangiitis (Churg-Strauss syndrome)	CTV3	X705i	Churg-Strauss vasculitis	-	-
Eosinophilic oesophagitis	SNOMED	235599003	Eosinophilic oesophagitis	Active	2006-01-31
Eosinophilic oesophagitis	SNOMED	721612007	Neonatal eosinophilic oesophagitis	Active	2017-01-31
Eosinophilic oesophagitis	SNOMED	735455001	Eosinophilic oesophagitis caused by food	Active	2018-01-31
Eosinophilic oesophagitis	SNOMED	770592009	Proton pump inhibitor responsive eosinophilic oesophagitis	Active	2019-01-31
Eosinophilic oesophagitis	SNOMED	938281000000104	Eosinophilic oesophagitis	Inactive	2014-10-01
Eosinophilic oesophagitis	CTV3	X3009	Eosinophilic oesophagitis	-	-
Fibrodysplasia ossificans progressiva	SNOMED	82725007	Progressive myositis ossificans	Active	2015-07-31
Fibrodysplasia ossificans progressiva	CTV3	N2311	Myositis ossificans progressiv | Myositis ossificans progressiva	-	-
Fibrodysplasia ossificans progressiva	CTV3	X7095	Hereditary myositis ossificans	-	-
Gaucher's disease	SNOMED	190794006	Gaucher's disease	Active	2002-01-31
Gaucher's disease	SNOMED	5963005	Subacute neuronopathic Gaucher's disease	Active	2002-01-31
Gaucher's disease	SNOMED	192791009	Cerebral degeneration in Gaucher's disease	Active	2006-01-31
Gaucher's disease	SNOMED	870313002	Perinatal lethal Gaucher disease	Active	2020-07-31
Gaucher's disease	SNOMED	1156813002	Gaucher disease with ophthalmoplegia and cardiovascular calcification	Active	2021-07-31
Gaucher's disease	SNOMED	62201009	Chronic non-neuropathic Gaucher's disease	Active	2021-07-31
Gaucher's disease	SNOMED	12246008	Acute neuronopathic Gaucher's disease	Active	2021-07-31
Gaucher's disease	SNOMED	1156792000	Atypical Gaucher disease due to saposin C deficiency	Active	2021-07-31
Gaucher's disease	SNOMED	2859005	Gaucher's disease	Inactive	2002-01-31
Gaucher's disease	CTV3	C3271	Glucosylceram b-glucosidas def | Glucosylceramide beta-glucosidase deficiency	-	-
Gaucher's disease	CTV3	X40VE	Glucocerebrosidase def typ III | Glucocerebrosidase deficiency type III	-	-
Gaucher's disease	CTV3	X40VF	Glucocerebrosidase def type I | Glucocerebrosidase deficiency type I	-	-
Gaucher's disease	CTV3	X40VD	Glucocerebrosidase def type II | Glucocerebrosidase deficiency type II	-	-
Gaucher's disease	CTV3	F1020	Cerebral degen.+Gaucher's dis | Cerebral degeneration in Gaucher's disease	-	-
Good syndrome	SNOMED	9893005	Immunodeficiency with thymoma	Active	2002-01-31

Disease	Ontology	Concept ID	Preferred term	Active Status	Date of last status change
Hereditary angioedema	SNOMED	234620006	Hereditary C1 esterase inhibitor deficiency - dysfunctional factor	Active	2002-01-31
Hereditary angioedema	SNOMED	234619000	Hereditary C1 esterase inhibitor deficiency - deficient factor	Active	2002-01-31
Hereditary angioedema	SNOMED	427167008	Hereditary angioneurotic oedema with normal C1 esterase inhibitor activity	Active	2007-07-31
Hereditary angioedema	SNOMED	82966003	Hereditary angioneurotic oedema	Active	2020-07-31
Hereditary angioedema	SNOMED	1230015008	Hereditary angioedema with C1Inh (C1 esterase inhibitor) deficiency	Active	2022-05-31
Hereditary angioedema	CTV3	X20IK	Her C1 ester inhib-dysf factor | Hereditary C1 esterase inhib defic - dysfunctional factor | Hereditary C1 esterase inhibitor deficiency - dysfunctional factor	-	-
Hereditary angioedema	CTV3	X20IJ	Her C1 ester inhib-def factor | Hereditary C1 esterase inhibitor defic - deficient factor | Hereditary C1 esterase inhibitor deficiency - deficient factor	-	-
Hereditary angioedema	CTV3	C3760	C1 esterase inhibitor defic | C1 esterase inhibitor deficiency	-	-
Hereditary haemorrhagic telangiectasia	SNOMED	21877004	Osler haemorrhagic telangiectasia syndrome	Active	2002-01-31
Hereditary haemorrhagic telangiectasia	SNOMED	1149069001	Juvenile polyposis syndrome with hereditary haemorrhagic telangiectasia	Active	2021-07-31
Hereditary haemorrhagic telangiectasia	SNOMED	1197033002	Hereditary haemorrhagic telangiectasia of gingiva	Active	2022-02-28
Hereditary haemorrhagic telangiectasia	SNOMED	155449008	Telangiectasia (& [hereditary haemorrhagic]) or diseases of capillaries NOS	Inactive	2002-01-31
Hereditary haemorrhagic telangiectasia	SNOMED	266324004	Telangiectasia (& [hereditary haemorrhagic]) or diseases of capillaries NOS	Inactive	2002-01-31
Hereditary haemorrhagic telangiectasia	CTV3	G770.	Hered haemorrhagic telangiect | Hereditary haemorrhagic telangiectasia	-	-
Hereditary haemorrhagic telangiectasia	CTV3	XE0XK	Telangiectasia (& [hered hae++ | Telangiectasia (& [hered haemorr]) or disease of capill NOS | Telangiectasia (& [hereditary haemorrhagic]) or diseases of capillaries NOS	-	-
Hypophosphatasia	SNOMED	20756002	Adult hypophosphatasia	Active	2002-01-31
Hypophosphatasia	SNOMED	30174008	Childhood hypophosphatasia	Active	2002-01-31
Hypophosphatasia	SNOMED	55236002	Infantile hypophosphatasia	Active	2002-01-31
Hypophosphatasia	SNOMED	190859005	Hypophosphatasia	Active	2002-01-31
Hypophosphatasia	SNOMED	190860000	Hypophosphatasia rickets	Active	2002-01-31
Hypophosphatasia	SNOMED	708672004	Odontohypophosphatasia	Active	2015-07-31
Hypophosphatasia	SNOMED	1184704003	Periodontitis exacerbated by hypophosphatasia	Active	2021-11-30
Hypophosphatasia	SNOMED	70848009	Hypophosphatasia	Inactive	2002-01-31
Hypophosphatasia	SNOMED	709556009	Periodontitis co-occurrent with hypophosphatasia	Inactive	2021-11-30
Hypophosphatasia	CTV3	X40Qk	Adult hypophosphatasia	-	-
Hypophosphatasia	CTV3	X40Qj	Childhood hypophosphatasia	-	-
Hypophosphatasia	CTV3	X40Qi	Infantile hypophosphatasia	-	-
Hypophosphatasia	CTV3	C3530	Hypophosphatasia	-	-
Hypophosphatasia	CTV3	C3531	Hypophosphatasia rickets	-	-
Myotonic dystrophy	SNOMED	77956009	Steinert myotonic dystrophy syndrome	Active	2002-01-31
Myotonic dystrophy	SNOMED	195031006	Cardiomyopathy in myotonic dystrophy	Active	2002-01-31
Myotonic dystrophy	SNOMED	715317001	Myotonic dystrophy type 2	Active	2016-07-31
Myotonic dystrophy	SNOMED	2816000	Dilated cardiomyopathy due to myotonic dystrophy	Active	2021-07-31
Myotonic dystrophy	SNOMED	240104008	Congenital myotonic dystrophy	Active	2021-10-31
Myotonic dystrophy	SNOMED	1177122009	Myotonic dystrophy	Active	2021-10-31
Myotonic dystrophy	SNOMED	108931000000102	Myotonic dystrophy	Inactive	2004-01-31
Myotonic dystrophy	CTV3	F3920	Myotonic dystrophy	-	-
Myotonic dystrophy	CTV3	XE18b	Myotonic disorders (& [dystr++ | Myotonic disorders (& [dystrophia myotonica])	-	-
Myotonic dystrophy	CTV3	G5581	Myotonic dystrophy cardiomyop. | Cardiomyopathy in myotonic dystrophy	-	-
Myotonic dystrophy	CTV3	X709b	Congenital myotonic dystrophy	-	-
Myotonic dystrophy	CTV3	XaetB	Proximal myotonic myopathy	-	-
Narcolepsy	SNOMED	60380001	Narcolepsy	Active	2002-01-31
Narcolepsy	SNOMED	193042000	Cataplexy and narcolepsy	Active	2002-01-31
Narcolepsy	SNOMED	91521000119104	Narcolepsy without cataplexy	Active	2013-07-31
Narcolepsy	SNOMED	735676003	Narcolepsy type 1	Active	2018-01-31
Narcolepsy	SNOMED	193043005	Cataplexy or narcolepsy NOS	Inactive	2010-01-31
Narcolepsy	SNOMED	654361000000105	Cataplexy or narcolepsy NOS	Inactive	2012-04-01
Narcolepsy	CTV3	F271.	Narcoleptic syndrome	-	-
Narcolepsy	CTV3	F27z.	Cataplexy or narcolepsy NOS	-	-
Narcolepsy	CTV3	F27..	Cataplexy and narcolepsy	-	-
Niemann-Pick disease, type C	SNOMED	86444004	Niemann-Pick disease, type C, acute form	Active	2002-01-31
Niemann-Pick disease, type C	SNOMED	72488000	Niemann-Pick disease, type C, chronic form	Active	2002-01-31
Niemann-Pick disease, type C	SNOMED	67855008	Niemann-Pick disease, type C, subacute form	Active	2002-01-31
Niemann-Pick disease, type C	SNOMED	66751000	Niemann-Pick disease, type C	Active	2002-01-31
Niemann-Pick disease, type C	SNOMED	86444004	Niemann-Pick disease, type C, acute form	Active	2002-01-31
Niemann-Pick disease, type C	SNOMED	72488000	Niemann-Pick disease, type C, chronic form	Active	2002-01-31
Niemann-Pick disease, type C	SNOMED	67855008	Niemann-Pick disease, type C, subacute form	Active	2002-01-31

Disease	Ontology	Concept ID	Preferred term	Active Status	Date of last status change
Niemann-Pick disease, type C	SNOMED	66751000	Niemann-Pick disease, type C	Active	2002-01-31
Niemann-Pick disease, type C	SNOMED	58459009	Sphingomyelin/cholesterol lipidosis	Active	2002-07-31
Niemann-Pick disease, type C	SNOMED	192792002	Cerebral degeneration in Niemann-Pick disease	Active	2006-01-31
Niemann-Pick disease, type C	SNOMED	1260366004	Dystonia due to Niemann-Pick disease type C	Active	2023-02-28
Niemann-Pick disease, type C	CTV3	X40VR	Niemann-Pick disease type C	-	-
Niemann-Pick disease, type C	CTV3	X40VR	Niemann-Pick disease type C	-	-
Osteogenesis imperfecta	SNOMED	7134007	Osteogenesis imperfecta, dominant perinatal lethal	Active	2002-01-31
Osteogenesis imperfecta	SNOMED	15552004	Osteogenesis imperfecta, recessive perinatal lethal, with microcephaly AND cataracts	Active	2002-01-31
Osteogenesis imperfecta	SNOMED	63890001	Osteogenesis imperfecta with blue sclerae AND dentinogenesis imperfecta	Active	2002-01-31
Osteogenesis imperfecta	SNOMED	64404003	Osteogenesis imperfecta with blue sclerae AND normal teeth	Active	2002-01-31
Osteogenesis imperfecta	SNOMED	86470003	Osteogenesis imperfecta, recessive perinatal lethal	Active	2002-01-31
Osteogenesis imperfecta	SNOMED	205496008	Osteogenesis imperfecta, perinatal lethal	Active	2002-01-31
Osteogenesis imperfecta	SNOMED	205497004	Osteogenesis imperfecta with normal sclerae, dominant form	Active	2002-01-31
Osteogenesis imperfecta	SNOMED	254110009	Osteogenesis imperfecta type IIA	Active	2002-01-31
Osteogenesis imperfecta	SNOMED	254111008	Osteogenesis imperfecta type IIB	Active	2002-01-31
Osteogenesis imperfecta	SNOMED	279309008	Osteogenesis imperfecta, type IV B	Active	2002-01-31
Osteogenesis imperfecta	SNOMED	280159008	Osteogenesis imperfecta, type IV A	Active	2002-01-31
Osteogenesis imperfecta	SNOMED	234968002	Dentinogenesis imperfecta - Shield's type I	Active	2002-01-31
Osteogenesis imperfecta	SNOMED	385482004	Osteogenesis imperfecta type I	Active	2003-01-31
Osteogenesis imperfecta	SNOMED	385483009	Osteogenesis imperfecta type III	Active	2003-01-31
Osteogenesis imperfecta	SNOMED	722110003	Osteogenesis imperfecta, retinopathy, seizures, intellectual disability syndrome	Active	2017-01-31
Osteogenesis imperfecta	SNOMED	733457006	Ehlers-Danlos and osteogenesis imperfecta syndrome	Active	2017-07-31
Osteogenesis imperfecta	SNOMED	78314001	Osteogenesis imperfecta	Active	2019-07-31
Osteogenesis imperfecta	SNOMED	782781006	High bone mass osteogenesis imperfecta	Active	2019-07-31
Osteogenesis imperfecta	SNOMED	1003379004	Osteogenesis imperfecta type 5	Active	2021-01-31
Osteogenesis imperfecta	SNOMED	1197018005	Osteogenesis imperfecta type IIC	Active	2022-02-28
Osteogenesis imperfecta	SNOMED	50937002	Osteogenesis imperfecta with normal sclerae, dominant form	Inactive	2002-01-31
Osteogenesis imperfecta	SNOMED	78354004	Osteogenesis imperfecta, perinatal lethal	Inactive	2002-01-31
Osteogenesis imperfecta	SNOMED	205492005	(Osteogenesis imperfecta) or (Vrolik's disease) or (syndromes: [Adair-Dighton] or [Lobstein's] or [Van der Hoeve's])	Inactive	2002-01-31
Osteogenesis imperfecta	SNOMED	205493000	Osteogenesis imperfecta	Inactive	2002-01-31
Osteogenesis imperfecta	SNOMED	254105005	Osteogenesis imperfecta	Inactive	2002-01-31
Osteogenesis imperfecta	SNOMED	254106006	Osteogenesis imperfecta	Inactive	2002-01-31
Osteogenesis imperfecta	SNOMED	254107002	Osteogenesis imperfecta	Inactive	2002-01-31
Osteogenesis imperfecta	SNOMED	254108007	Osteogenesis imperfecta	Inactive	2002-01-31
Osteogenesis imperfecta	SNOMED	254109004	Osteogenesis imperfecta	Inactive	2002-01-31
Osteogenesis imperfecta	SNOMED	3508009	Osteogenesis imperfecta with blue sclerae	Inactive	2003-01-31
Osteogenesis imperfecta	SNOMED	54625007	Osteogenesis imperfecta with progressive deformity AND normal sclerae	Inactive	2003-01-31
Osteogenesis imperfecta	SNOMED	205495007	Osteogenesis imperfecta - unclassifiable	Inactive	2003-01-31
Osteogenesis imperfecta	SNOMED	278454001	Fragilitas ossium congenita	Inactive	2003-01-31
Osteogenesis imperfecta	SNOMED	205498009	Osteogenesis imperfecta NOS	Inactive	2010-01-31
Osteogenesis imperfecta	SNOMED	657741000000102	Osteogenesis imperfecta NOS	Inactive	2012-04-01
Osteogenesis imperfecta	CTV3	PG51.	(Osteogen imperf)(Vrolik dis++ | (Osteogen imperf)(Vrolik dis)(syn:[Adair-D][Lobst][V Hoeve]) | (Osteogenesis imperfecta) or (Vrolik's disease) or (syndromes: [Adair-Dighton] or [Lobstein's] or [Van der Hoeve's])	-	-
Osteogenesis imperfecta	CTV3	PG511	Osteopsathyrosis	-	-
Osteogenesis imperfecta	CTV3	PG512	Osteogen imperfecta - unclass | Osteogenesis imperfecta - unclassifiable	-	-
Osteogenesis imperfecta	CTV3	PG51z	Osteogenesis imperfecta NOS	-	-
Osteogenesis imperfecta	CTV3	XE1MD	Osteogenesis imperfecta	-	-
Osteogenesis imperfecta	CTV3	PG514	Osteogenes imperfecta type II | Osteogenesis imperfecta type II	-	-
Osteogenesis imperfecta	CTV3	PG516	Osteogenes imperfecta type IV | Osteogenesis imperfecta type IV	-	-
Osteogenesis imperfecta	CTV3	X78BA	Osteogenes imperfecta type IIA | Osteogenesis imperfecta type IIA	-	-
Osteogenesis imperfecta	CTV3	X78BB	Osteogenes imperfecta type IIB | Osteogenesis imperfecta type IIB	-	-
Osteogenesis imperfecta	CTV3	PG513	Osteogenesis imperfecta type I	-	-
Osteogenesis imperfecta	CTV3	X00kq	van de Hoeve syndrome	-	-
Osteogenesis imperfecta	CTV3	PG515	Osteogenesis imperfecta III	-	-
Osteogenesis imperfecta	CTV3	X20P1	Dentinogen imp Shield's type I | Dentinogenesis imperfecta - Shield's type I	-	-
PTEN hamartoma tumour syndrome	SNOMED	23150001	Proteus syndrome	Active	2002-01-31
PTEN hamartoma tumour syndrome	SNOMED	58037000	Cowden syndrome	Active	2002-01-31
PTEN hamartoma tumour syndrome	SNOMED	234138005	Bannayan syndrome	Active	2002-01-31

Disease	Ontology	Concept ID	Preferred term	Active Status	Date of last status change
PTEN hamartoma tumour syndrome	SNOMED	23150001	Proteus syndrome	Active	2002-01-31
PTEN hamartoma tumour syndrome	SNOMED	58037000	Cowden syndrome	Active	2002-01-31
PTEN hamartoma tumour syndrome	SNOMED	234138005	Bannayan syndrome	Active	2002-01-31
PTEN hamartoma tumour syndrome	SNOMED	716862002	Proteus like syndrome	Active	2016-07-31
PTEN hamartoma tumour syndrome	SNOMED	722859001	PTEN hamartoma tumour syndrome	Active	2017-01-31
PTEN hamartoma tumour syndrome	SNOMED	722859001	PTEN hamartoma tumour syndrome	Active	2017-01-31
PTEN hamartoma tumour syndrome	SNOMED	763867001	Segmental outgrowth, lipomatosis, arteriovenous malformation, epidermal naevus syndrome	Active	2018-07-31
PTEN hamartoma tumour syndrome	SNOMED	763867001	Segmental outgrowth, lipomatosis, arteriovenous malformation, epidermal naevus syndrome	Active	2018-07-31
PTEN hamartoma tumour syndrome	CTV3	X207l	Proteus syndrome	-	-
PTEN hamartoma tumour syndrome	CTV3	X50H3	Cowden syndrome	-	-
PTEN hamartoma tumour syndrome	CTV3	X207k	Bannayan syndrome	-	-
PTEN hamartoma tumour syndrome	CTV3	X207l	Proteus syndrome	-	-
PTEN hamartoma tumour syndrome	CTV3	X50H3	Cowden syndrome	-	-
PTEN hamartoma tumour syndrome	CTV3	X207k	Bannayan syndrome	-	-
Paroxysmal nocturnal haemoglobinuria	SNOMED	1963002	Paroxysmal nocturnal haemoglobinuria	Active	2002-01-31
Paroxysmal nocturnal haemoglobinuria	CTV3	D1121	Paroxy nocturn haemoglobinuria | Paroxysmal nocturnal haemoglobinuria	-	-
Peutz-Jeghers syndrome	SNOMED	54411001	Peutz-Jeghers syndrome	Active	2002-01-31
Peutz-Jeghers syndrome	SNOMED	53633000	Peutz-Jeghers polyps of small bowel	Active	2002-01-31
Peutz-Jeghers syndrome	SNOMED	277161008	Peutz Jeghers polyp	Active	2002-01-31
Peutz-Jeghers syndrome	SNOMED	157029009	Peutz-Jeghers syndrome	Inactive	2002-01-31
Peutz-Jeghers syndrome	CTV3	PK60.	Peutz-Jeghers syndrome	-	-
Peutz-Jeghers syndrome	CTV3	Xa0KK	Peutz Jehgers polyp	-	-
Prader-Willi syndrome	SNOMED	89392001	Prader-Willi syndrome	Active	2002-01-31
Prader-Willi syndrome	SNOMED	89392001	Prader-Willi syndrome	Active	2002-01-31
Prader-Willi syndrome	SNOMED	205794007	(Multiple system congenital anomalies NEC) or (Prader-Willi syndrome) or (Noonan's syndrome)	Inactive	2002-01-31
Prader-Willi syndrome	SNOMED	205794007	(Multiple system congenital anomalies NEC) or (Prader-Willi syndrome) or (Noonan's syndrome)	Inactive	2002-01-31
Prader-Willi syndrome	SNOMED	99311000000109	Prader-Willi syndrome	Inactive	2004-01-31
Prader-Willi syndrome	SNOMED	99311000000109	Prader-Willi syndrome	Inactive	2004-01-31
Prader-Willi syndrome	CTV3	PKy93	Prader-Willi syndrome	-	-
Prader-Willi syndrome	CTV3	PKy0.	(Multi syst cong anom NEC) o++ | (Multi syst cong anom NEC) or (Prader-Willi) or (Noonan syn) | (Multiple system congenital anomalies NEC) or (Prader-Willi syndrome) or (Noonan's syndrome)	-	-
Prader-Willi syndrome	CTV3	PKy93	Prader-Willi syndrome	-	-
Prader-Willi syndrome	CTV3	PKy0.	(Multi syst cong anom NEC) o++ | (Multi syst cong anom NEC) or (Prader-Willi) or (Noonan syn) | (Multiple system congenital anomalies NEC) or (Prader-Willi syndrome) or (Noonan's syndrome)	-	-
SAPHO syndrome	SNOMED	60684003	SAPHO syndrome	Active	2002-01-31
SAPHO syndrome	SNOMED	203140009	Synovitis, acne, pustulosis palmaris, hyperostosis, osteomyelitis syndrome	Inactive	2002-01-31
SAPHO syndrome	CTV3	X702V	Syno,acne,pust palm,hypost,om | Synovitis,acne,pustulosis palmaris,hyperostos,osteomyel synd | Synovitis, acne, pustulosis palmaris, hyperostosis, osteomyelitis syndrome	-	-
Sturge-Weber syndrome	SNOMED	19886006	Sturge-Weber syndrome	Active	2002-01-31
Sturge-Weber syndrome	SNOMED	1172358003	Gingival enlargement due to Sturge-Weber syndrome	Active	2021-09-30
Sturge-Weber syndrome	SNOMED	157030004	Sturge-Weber syndrome	Inactive	2002-01-31
Sturge-Weber syndrome	CTV3	PK61.	Sturge-Weber syndrome	-	-
Tuberous sclerosis	SNOMED	7199000	Tuberous sclerosis syndrome	Active	2002-01-31
Tuberous sclerosis	SNOMED	36025004	Fibrous skin tumour of tuberous sclerosis	Active	2002-01-31
Tuberous sclerosis	SNOMED	233718008	Pulmonary tuberous sclerosis	Active	2002-01-31
Tuberous sclerosis	SNOMED	254243001	Ash leaf spot, tuberous sclerosis	Active	2002-01-31
Tuberous sclerosis	SNOMED	403823001	Periungual fibroma in tuberous sclerosis	Active	2005-07-31
Tuberous sclerosis	SNOMED	707433009	Lymphangioleiomyomatosis due to tuberous sclerosis syndrome	Active	2015-01-31
Tuberous sclerosis	SNOMED	765331004	Autosomal dominant polycystic kidney disease type 1 with tuberous sclerosis	Active	2018-07-31
Tuberous sclerosis	SNOMED	157027006	Tuberous sclerosis	Inactive	2002-01-31
Tuberous sclerosis	CTV3	PK5..	Tuberous sclerosis	-	-
Tuberous sclerosis	CTV3	X78E9	Adenoma sebaceum	-	-
Tuberous sclerosis	CTV3	X102q	Pulmonary tuberose sclerosis	-	-
Tuberous sclerosis	CTV3	X78E7	Ash leaf spot, tub sclerosis | Ash leaf spot, tuberous sclerosis	-	-
Turner syndrome	SNOMED	38804009	Turner syndrome	Active	2002-01-31
Turner syndrome	SNOMED	205686009	Karyotype 46, X iso (Xq)	Active	2002-01-31
Turner syndrome	SNOMED	205687000	Karyotype 46, X with abnormal sex chromosome except iso (Xq)	Active	2002-01-31
Turner syndrome	SNOMED	254281006	Turner's phenotype - ring chromosome karyotype	Active	2002-01-31
Turner syndrome	SNOMED	254280007	Turner's phenotype, partial X deletion karyotype	Active	2002-01-31
Turner syndrome	SNOMED	38804009	Turner syndrome	Active	2002-01-31

Disease	Ontology	Concept ID	Preferred term	Active Status	Date of last status change
Turner syndrome	SNOMED	205686009	Karyotype 46, X iso (Xq)	Active	2002-01-31
Turner syndrome	SNOMED	205687000	Karyotype 46, X with abnormal sex chromosome except iso (Xq)	Active	2002-01-31
Turner syndrome	SNOMED	254281006	Turner's phenotype - ring chromosome karyotype	Active	2002-01-31
Turner syndrome	SNOMED	254280007	Turner's phenotype, partial X deletion karyotype	Active	2002-01-31
Turner syndrome	SNOMED	710008008	Monosomy X	Active	2015-07-31
Turner syndrome	SNOMED	710010005	Mosaic Turner syndrome	Active	2015-07-31
Turner syndrome	SNOMED	710008008	Monosomy X	Active	2015-07-31
Turner syndrome	SNOMED	710010005	Mosaic Turner syndrome	Active	2015-07-31
Turner syndrome	SNOMED	268356004	(Gonadal dysgenesis (& Turner)) or Turner's syndrome	Inactive	2002-01-31
Turner syndrome	SNOMED	157020008	(Gonadal dysgenesis (& Turner)) or Turner's syndrome	Inactive	2002-01-31
Turner syndrome	SNOMED	268356004	(Gonadal dysgenesis (& Turner)) or Turner's syndrome	Inactive	2002-01-31
Turner syndrome	SNOMED	157020008	(Gonadal dysgenesis (& Turner)) or Turner's syndrome	Inactive	2002-01-31
Turner syndrome	SNOMED	205993006	[X]Other variants of Turner's syndrome	Inactive	2009-01-31
Turner syndrome	SNOMED	205993006	[X]Other variants of Turner's syndrome	Inactive	2009-01-31
Turner syndrome	SNOMED	268299006	Turner's syndrome NOS	Inactive	2010-01-31
Turner syndrome	SNOMED	268299006	Turner's syndrome NOS	Inactive	2010-01-31
Turner syndrome	SNOMED	597191000000108	Turner's syndrome NOS	Inactive	2012-04-01
Turner syndrome	SNOMED	597191000000108	Turner's syndrome NOS	Inactive	2012-04-01
Turner syndrome	SNOMED	205688005	Turner's phenotype, mosaicism 45, X; 46, XX or 45, X; 46, XY	Inactive	2015-07-31
Turner syndrome	SNOMED	205688005	Turner's phenotype, mosaicism 45, X; 46, XX or 45, X; 46, XY	Inactive	2015-07-31
Turner syndrome	SNOMED	440171000000109	[X]Other variants of Turner's syndrome	Inactive	2015-10-01
Turner syndrome	SNOMED	440171000000109	[X]Other variants of Turner's syndrome	Inactive	2015-10-01
Turner syndrome	CTV3	.N722	NA	-	-
Turner syndrome	CTV3	PJ63.	Turner's syndrome	-	-
Turner syndrome	CTV3	PJ636	Turner's phenotype: [variant++ | Turner's phenotype: [variant karyotypes] | Turner's phenotype: [other variant karyotypes] or [ring chromosome karyotype] or [partial X deletion karyotype]	-	-
Turner syndrome	CTV3	PJ63z	(Turner syn NOS) or (Bonn-Ul++ | (Turner syn NOS) or (Bonn-Ullr syn NOS) or (Ovar dwarf NEC) | (Turner's syndrome NOS) or (Bonnevie-Ullrich syndrome NOS) or (Ovarian dwarfism NEC)	-	-
Turner syndrome	CTV3	PyuA5	[X]Oth variants Turner's synd | [X]Other variants of Turner's syndrome	-	-
Turner syndrome	CTV3	X78Ey	Ovarian dwarfism NEC	-	-
Turner syndrome	CTV3	X78Ez	Bonnevie-Ullrich syndrome NOS	-	-
Turner syndrome	CTV3	XE1Me	Turner's, other variant karyo | Turner's phenotype, other variant karyotypes	-	-
Turner syndrome	CTV3	XE1Mf	Turner's syndrome NOS	-	-
Turner syndrome	CTV3	XE1OW	(Gonadal dysgenesis (& Turne++ | (Gonadal dysgenesis (& Turner)) or Turner's syndrome	-	-
Turner syndrome	CTV3	PJ631	Karyotype 45, X	-	-
Turner syndrome	CTV3	PJ634	Turn mosa 45X/46XX or 45X/46XY | Turner's phenotype, mosaicism 45, X; 46, XX or 45, X; 46, XY	-	-
Turner syndrome	CTV3	.N722	NA	-	-
Turner syndrome	CTV3	PJ63.	Turner's syndrome	-	-
Turner syndrome	CTV3	PJ636	Turner's phenotype: [variant++ | Turner's phenotype: [variant karyotypes] | Turner's phenotype: [other variant karyotypes] or [ring chromosome karyotype] or [partial X deletion karyotype]	-	-
Turner syndrome	CTV3	PJ63z	(Turner syn NOS) or (Bonn-Ul++ | (Turner syn NOS) or (Bonn-Ullr syn NOS) or (Ovar dwarf NEC) | (Turner's syndrome NOS) or (Bonnevie-Ullrich syndrome NOS) or (Ovarian dwarfism NEC)	-	-
Turner syndrome	CTV3	PyuA5	[X]Oth variants Turner's synd | [X]Other variants of Turner's syndrome	-	-
Turner syndrome	CTV3	X78Ey	Ovarian dwarfism NEC	-	-
Turner syndrome	CTV3	X78Ez	Bonnevie-Ullrich syndrome NOS	-	-
Turner syndrome	CTV3	XE1Me	Turner's, other variant karyo | Turner's phenotype, other variant karyotypes	-	-
Turner syndrome	CTV3	XE1Mf	Turner's syndrome NOS	-	-
Turner syndrome	CTV3	XE1OW	(Gonadal dysgenesis (& Turne++ | (Gonadal dysgenesis (& Turner)) or Turner's syndrome	-	-
Turner syndrome	CTV3	PJ631	Karyotype 45, X	-	-
Turner syndrome	CTV3	PJ634	Turn mosa 45X/46XX or 45X/46XY | Turner's phenotype, mosaicism 45, X; 46, XX or 45, X; 46, XY	-	-
Williams syndrome	SNOMED	63247009	Williams syndrome	Active	2002-01-31
Williams syndrome	CTV3	PKy4.	William syndrome	-	-
X-linked agammaglobulinaemia	SNOMED	65880007	X-linked agammaglobulinaemia	Active	2002-01-31
X-linked agammaglobulinaemia	SNOMED	234533006	X-linked agammaglobulinaemia with growth hormone deficiency	Active	2002-01-31
X-linked agammaglobulinaemia	SNOMED	65880007	X-linked agammaglobulinaemia	Active	2002-01-31
X-linked agammaglobulinaemia	SNOMED	234533006	X-linked agammaglobulinaemia with growth hormone deficiency	Active	2002-01-31
X-linked agammaglobulinaemia	SNOMED	190983003	Hypogammaglobulinaemia: [congenital] or [agammaglobulinaemia: (Bruton's) or (congenital sex-linked & [X-linked])]	Inactive	2002-01-31
X-linked agammaglobulinaemia	SNOMED	190983003	Hypogammaglobulinaemia: [congenital] or [agammaglobulinaemia: (Bruton's) or (congenital sex-linked & [X-linked])]	Inactive	2002-01-31
X-linked agammaglobulinaemia	CTV3	X20Gc	X-linked agammaglobulinaemia	-	-

Disease	Ontology	Concept ID	Preferred term	Active Status	Date of last status change
X-linked agammaglobulinaemia	CTV3	C3905	Hypogammaglob:[cong][agammag++ | Hypogammaglob:[cong][agammaglob:(Brut)(cong sex-link & [X])] | Hypogammaglobulinaemia: [congenital] or [agammaglobulinaemia: (Bruton's) or (congenital sex-linked & [X-linked])]	-	-
X-linked agammaglobulinaemia	CTV3	X20Gd	X-link agammaglob+grth hor def | X-linked agammaglobulinaemia with growth hormone deficiency	-	-
X-linked agammaglobulinaemia	CTV3	X20Gc	X-linked agammaglobulinaemia	-	-
X-linked agammaglobulinaemia	CTV3	C3905	Hypogammaglob:[cong][agammag++ | Hypogammaglob:[cong][agammaglob:(Brut)(cong sex-link & [X])] | Hypogammaglobulinaemia: [congenital] or [agammaglobulinaemia: (Bruton's) or (congenital sex-linked & [X-linked])]	-	-
X-linked agammaglobulinaemia	CTV3	X20Gd	X-link agammaglob+grth hor def | X-linked agammaglobulinaemia with growth hormone deficiency	-	-
X-linked hypophosphataemia	SNOMED	82236004	Familial x-linked hypophosphataemic vitamin D refractory rickets	Active	2002-01-31
X-linked hypophosphataemia	SNOMED	82236004	Familial x-linked hypophosphataemic vitamin D refractory rickets	Active	2002-01-31
X-linked hypophosphataemia	SNOMED	855451000000106	X-linked hypophosphataemic rickets	Inactive	2012-10-01
X-linked hypophosphataemia	CTV3	C3532	Vitamin D-resistant rickets	-	-
X-linked hypophosphataemia	CTV3	X40Qq	X-lnkd hypophosphataem rickets | X-linked hypophosphataemic rickets	-	-
X-linked hypophosphataemia	CTV3	X40RH	Familial hypophosphataemia	-	-
X-linked hypophosphataemia	CTV3	Xa1As	Fam hypophosphataemic rickets | Familial hypophosphataemic rickets	-	-
X-linked hypophosphataemia	CTV3	C3532	Vitamin D-resistant rickets	-	-
X-linked hypophosphataemia	CTV3	X40Qq	X-lnkd hypophosphataem rickets | X-linked hypophosphataemic rickets	-	-
X-linked hypophosphataemia	CTV3	X40RH	Familial hypophosphataemia	-	-
X-linked hypophosphataemia	CTV3	Xa1As	Fam hypophosphataemic rickets | Familial hypophosphataemic rickets	-	-
