# Supplementary material for: Evaluating algorithmic approaches to rare disease case-finding: a retrospective validation study using electronic health records
Source: Orphanet J Rare Dis. 2026 Feb 4;21:120. doi: 10.1186/s13023-026-04240-6 (PMC13041464; doi:10.1186/s13023-026-04240-6)
Supplement: Supplementary file 2 — Supplementary Material 2 [file 13023_2026_4240_MOESM2_ESM.pdf]

Additional file 2

Prevalence figures used with references

Disease	Prevalence per 100,000	Reference
Hereditary angioedema	3.2	Hereditary Angioedema: A Review. Zafra H. WMJ. 2022 Apr;121(1):48-53.
PTEN hamartoma tumour syndrome	0.4	Novel PTEN mutations in patients with Cowden disease: absence of clear genotype-phenotype correlations. Nelen MR, Kremer H, Konings IB, Schoute F, van Essen AJ, Koch R, Woods CG, Fryns JP, Hamel B, Hoefsloot LH, Peeters EA, Padberg GW. Eur J Hum Genet. 1999 Apr;7(3):267-7 doi: 10.1038/sj.ejhg.5200289.
Bardet-Biedl syndrome	0.7	Bardet-Biedl Syndrome-Multiple Kaleidoscope Images: Insight into Mechanisms of Genotype-Phenotype Correlations. Florea L, Caba L, Gorduza EV. Genes (Basel). 2021 Aug 29;12(9):1353. doi: 10.3390/genes12091353.
Alkaptonuria	0.7	Alkaptonuria. Sharabi AF, Goudar RB. 2023 Aug 8. In: StatPearls [Internet]. Treasure Island (FL): StatPearls Publishing; 2024 Jan–.
Beckwith-Wiedemann syndrome	8.65	Beckwith-Wiedemann Syndrome. Shuman C, Kalish JM, Weksberg R. 2000 Mar 3 [updated 2023 Sep 21]. In: Adam MP, Feldman J, Mirzaa GM, Pagon RA, Wallace SE, Amemiya A, editors. GeneReviews(®) [Internet]. Seattle (WA): University of Washington, Seattle; 1993–2024.
Williams syndrome	13.3	Williams syndrome. Kozel BA, Barak B, Kim CA, Mervis CB, Osborne LR, Porter M, Pober BR. Nat Rev Dis Primers. 2021 Jun 17;7(1):42. doi: 10.1038/s41572-021-00276-z.
Prader-Willi syndrome	6.67	Prader-Willi syndrome. Cassidy SB, Schwartz S, Miller JL, Driscoll DJ. Genet Med. 2012 Jan;14(1):10-26. doi: 10.1038/gim.0b013e31822bead0. Epub 2011 Sep 26.
X-linked agammaglobulinaemia	0.225	X-Linked Agammaglobulinemia. Smith CIE, Berglöf A. 2001 Apr 5 [updated 2024 Jun 27]. In: Adam MP, Feldman J, Mirzaa GM, Pagon RA, Wallace SE, Amemiya A, editors. GeneReviews(®) [Internet]. Seattle (WA): University of Washington, Seattle; 1993–2024.
Turner syndrome	25	Turner syndrome: diagnosis and management. Morgan T. Am Fam Physician. 2007 Aug 1;76(3):405-10.
Good syndrome	0.2	Good syndrome: an adult-onset immunodeficiency remarkable for its high incidence of invasive infections and autoimmune complications. Malphettes M, Gérard L, Galicier L, Boutboul D, Asli B, Szalat R, Perlat A, Masseau A, Schleinitz N, Le Guenno G, Viallard JF, Bonnotte B, Thiercelin-Legrand MF, Sanhes L, Borie R, Georgin-Lavialle S, Fieschi C, Oksenhendler E; DEFicit Immunitaire de l'adulte Study Group. Clin Infect Dis. 2015 Jul 15;61(2):e13-9. doi: 10.1093/cid/civ269. Epub 2015 Mar 31.
Narcolepsy	44.3	Prevalence of narcolepsy and other sleep disorders and frequency of diagnostic tests from 2013-2016 in insured patients actively seeking care. Acquavella J, Mehra R, Bron M, Suomi JM, Hess GP. J Clin Sleep Med. 2020 Aug 15;16(8):1255-1263. doi: 10.5664/jcsm.8482.
X-linked hypophosphataemia	1.4	Prevalence and Mortality of Individuals With X-Linked Hypophosphatemia: A United Kingdom Real-World Data Analysis. Hawley S, Shaw NJ, Delmestri A, Prieto-Alhambra D, Cooper C, Pinedo-Villanueva R, Javaid MK. J Clin Endocrinol Metab. 2020 Mar 1;105(3):e871-8. doi: 10.1210/clinem/dgz203.
Gaucher's disease	2	Consequences of diagnostic delays in type 1 Gaucher disease: the need for greater awareness among hematologists-oncologists and an opportunity for early diagnosis and intervention. Mistry PK, Sadan S, Yang R, Yee J, Yang M. Am J Hematol. 2007 Aug;82(8):697-701. doi: 10.1002/ajh.20908.
Common variable immunodeficiency	4	The burden of common variable immunodeficiency disorders: a retrospective analysis of the European Society for Immunodeficiency (ESID) registry data. Odnoletkova I, Kindle G, Quinti I, Grimbacher B, Knerr V, Gathmann B, Ehl S, Mahlaoui N, Van Wilder P, Bogaerts K, de Vries E; Plasma Protein Therapeutics Association (PPTA) Taskforce. Orphanet J Rare Dis. 2018 Nov 12;13(1):201. doi: 10.1186/s13023-018-0941-0.
Alpha-1-antitrypsin deficiency	35	A review of α1-antitrypsin deficiency. Stoller JK, Aboussouan LS. Am J Respir Crit Care Med. 2012 Feb 1;185(3):246-59. doi: 10.1164/rccm.201108-1428CI. Epub 2011 Sep 29.

Hereditary haemorrhagic telangiectasia	10.6	The UK prevalence of hereditary haemorrhagic telangiectasia and its association with sex, socioeconomic status and region of residence: a population-based study. Donaldson JW, McKeever TM, Hall IP, Hubbard RB, Fogarty AW. Thorax. 2014 Feb;69(2):161-7. doi: 10.1136/thoraxjnl-2013-203720. Epub 2013 Nov 4.
Wilson's disease	1.55	Estimating the clinical prevalence of Wilson's disease in the UK. Wijayasiri P, Hayre J, Nicholson ES, Kaye P, Wilkes EA, Evans J, Aithal GP, Jones G, Pearce F, Aravinthan AD. JHEP Rep. 2021 Jul 7;3(5):100329. doi: 10.1016/j.jhepr.2021.100329. eCollection 2021 Oct.
DiGeorge syndrome (22q11 deletion)	21	DiGeorge Syndrome. Lackey AE, Muzio MR. 2023 Aug 8. In: StatPearls [Internet]. Treasure Island (FL): StatPearls Publishing; 2024 Jan–.
Duchenne muscular dystrophy	4.8	Global prevalence of Duchenne and Becker muscular dystrophy: a systematic review and meta-analysis. Salari N, Fatahi B, Valipour E, Kazeminia M, Fatahian R, Kiaei A, Shohaimi S, Mohammadi M. J Orthop Surg Res. 2022 Feb 15;17(1):96. doi: 10.1186/s13018-022-02996-8.
SAPHO syndrome	10	Synovitis, acne, pustulosis, hyperostosis, and osteitis syndrome: review and update. Liu S, Tang M, Cao Y, Li C. Ther Adv Musculoskelet Dis. 2020 May 12;12:1759720X20912865. doi: 10.1177/1759720X20912865. eCollection 2020.
Myotonic dystrophy	22.9	The hallmarks of myotonic dystrophy type 1 muscle dysfunction. Ozimski LL, Sabater-Arcis M, Bargiela A, Artero R. Biol Rev Camb Philos Soc. 2021 Apr;96(2):716-730. doi: 10.1111/brv.12674. Epub 2020 Dec 2.
Peutz-Jeghers syndrome	2.18	Peutz-Jeghers Syndrome. McGarrity TJ, Amos CI, Baker MJ. 2001 Feb 23 [updated 2021 Sep 2]. In: Adam MP, Feldman J, Mirzaa GM, Pagon RA, Wallace SE, Amemiya A, editors. GeneReviews(®) [Internet]. Seattle (WA): University of Washington, Seattle; 1993–2024.
Eosinophilic oesophagitis	6.5	Eosinophilic Esophagitis. Roussel JM, Pandit S. 2023 Aug 8. In: StatPearls [Internet]. Treasure Island (FL): StatPearls Publishing; 2024 Jan–.
Eosinophilic granulomatosis with polyangiitis (Churg-Strauss syndrome)	1.235	Churg-Strauss syndrome. Greco A, Rizzo MI, De Virgilio A, Gallo A, Fusconi M, Ruoppolo G, Altissimi G, De Vincentiis M. Autoimmun Rev. 2015 Apr;14(4):341-8. doi: 10.1016/j.autrev.2014.12.004. Epub 2014 Dec 11.
Fibrodysplasia ossificans progressiva	0.07435	Liljesthr�m, M., Pignolo, R.J. and Kaplan, F.S. (2020) Epidemiology of the Global Fibrodysplasia Ossificans Progressiva (FOP) community, Journal of Rare Diseases Research & Treatment. Available at: https://www.rarediseasesjournal.com/articles/epidemiology-of-the-global-fibrodysplasia-ossificans-progressiva-fop-community.html
Hypophosphatasia	15.7	(2017) NHS choices. Available at: https://www.england.nhs.uk/2017/07/nhs-patients-with-rare-bone-disease-to-benefit-from-potentially-life-transforming-drug/
Tuberous sclerosis	5	Tuberous Sclerosis. Rout P, Zamora EA, Aeddula NR. 2024 Sep 3. In: StatPearls [Internet]. Treasure Island (FL): StatPearls Publishing; 2024 Jan–.
Behçet's disease	0.64	Behcet's disease: epidemiology, clinical manifestations, and diagnosis. Davatchi F, Chams-Davatchi C, Shams H, Shahram F, Nadji A, Akhlaghi M, Faezi T, Ghodsi Z, Sadeghi Abdollahi B, Ashofteh F, Mohtasham N, Kavosi H, Masoumi M. Expert Rev Clin Immunol. 2017 Jan;13(1):57-65. doi: 10.1080/1744666X.2016.1205486. Epub 2016 Jul 11.
Niemann-Pick disease, type C	0.745	Niemann-Pick disease type C. Vanier MT. Orphanet J Rare Dis. 2010 Jun 3;5:16. doi: 10.1186/1750-1172-5-16.
Sturge-Weber syndrome	3.5	Sturge-Weber Syndrome: A Review. Higueros E, Roe E, Granell E, Baselga E. Actas Dermosifiliogr. 2017 Jun;108(5):407-417. doi: 10.1016/j.ad.2016.09.022. Epub 2017 Jan 23.
Paroxysmal nocturnal haemoglobinuria	3.81	The incidence and prevalence of patients with paroxysmal nocturnal haemoglobinuria and aplastic anaemia PNH syndrome: A retrospective analysis of the UK's population-based haematological malignancy research network 2004-2018. Richards SJ, Painter D, Dickinson AJ, Griffin M, Munir T, Arnold L, Payne D, Pike A, Muus P, Hill A, Newton DJ, McKinley C, Jones R, Kelly R, Smith A, Roman E, Hillmen P. Eur J Haematol. 2021 Aug;107(2):211-218. doi: 10.1111/ejh.13640. Epub 2021 Jun 9.
Osteogenesis imperfecta	5.83	Osteogenesis Imperfecta. Subramanian S, Anastasopoulou C, Viswanathan VK. 2023 Feb 6. In: StatPearls [Internet]. Treasure Island (FL): StatPearls Publishing; 2024 Jan–.
Alström syndrome	0.55	Alström Syndrome. Paisey RB, Steeds R, Barrett T, Williams D, Geberhiwot T, Gunay-Aygun M. 2003 Feb 7 [updated 2019 Jun 13]. In: Adam MP, Feldman J, Mirzaa GM, Pagon RA, Wallace SE, Amemiya A, editors. GeneReviews(®) [Internet]. Seattle (WA): University of Washington, Seattle; 1993–2024.
Dermatomyositis	20.625	An overview of polymyositis and dermatomyositis. Findlay AR, Goyal NA, Mozaffar T. Muscle Nerve. 2015 May;51(5):638-56. doi: 10.1002/mus.24566.
