# Supplementary material for: Evaluating algorithmic approaches to rare disease case-finding: a retrospective validation study using electronic health records
Source: Orphanet J Rare Dis. 2026 Feb 4;21:120. doi: 10.1186/s13023-026-04240-6 (PMC13041464; doi:10.1186/s13023-026-04240-6)
Supplement: Supplementary file 3 — Supplementary Material 3 [file 13023_2026_4240_MOESM3_ESM.pdf]

Additional file 3: Algorithm performance metrics

Figures in brackets represent 95% confidence intervals. Positive predictive value (PPV) is provided with adjustment based on disease prevalence from literature and coded prevalence in the study population. For the columns indicating median months flagged relative to diagnosis, negative months indicate flagging occurred before diagnosis. N/A indicates that no patients were flagged in this group.

Algorithm	Sensitivity	Specificity	PPV (adjusted by literature prevalence)	PPV (adjusted by coded prevalence)	Positive likelihood ratio	Negative likelihood ratio	Percentage flagged pre-dx	Median months flagged relative to dx (of those flagged before dx)	Median months flagged relative to dx (of all flagged patients)
Alpha-1-antitrypsin deficiency (v1)	0.02% (0.00 - 0.11%)	99.9989% (99.9984 - 99.9993%)	0.64% (0.09 - 4.59%)	0.37% (0.05 - 2.70%)	18 (2 - 137)	1.00 (1.00 - 1.00)	0	N/A	328
Alpha-1-antitrypsin deficiency (v2)	4.40% (3.86 - 5.00%)	99.3213% (99.3098 - 99.3326%)	0.23% (0.20 - 0.26%)	0.13% (0.12 - 0.15%)	6 (6 - 7)	0.96 (0.96 - 0.97)	58.7	-75	-9
Alkaptonuria	0.91% (0.02 - 4.96%)	99.9925% (99.9912 - 99.9937%)	0.08% (0.01 - 0.60%)	0.05% (0.01 - 0.37%)	121 (17 - 858)	0.99 (0.97 - 1.01)	100	-5	-5
Alström syndrome	5.45% (1.14 - 15.12%)	99.9999% (99.9996 - 100.0000%)	23.08% (4.86 - 63.78%)	10.42% (1.94 - 40.57%)	54,545 (9,295 - 320,100)	0.95 (0.89 - 1.01)	0	N/A	13
Bardet-Biedl syndrome	13.62% (9.50 - 18.68%)	99.9999% (99.9996 - 100.0000%)	48.80% (18.68 - 79.82%)	55.66% (23.23 - 83.89%)	136,169 (32,820 - 564,956)	0.86 (0.82 - 0.91)	3.2	-30	110
Behçet's disease	3.15% (2.50 - 3.92%)	99.9925% (99.9913 - 99.9937%)	0.27% (0.21 - 0.35%)	4.22% (3.26 - 5.46%)	423 (323 - 554)	0.97 (0.96 - 0.98)	51.9	-15	0
Beckwith-Wiedemann syndrome	20.79% (17.16 - 24.80%)	99.9967% (99.9958 - 99.9974%)	35.27% (28.75 - 42.39%)	10.15% (7.72 - 13.23%)	6,299 (4,665 - 8,506)	0.79 (0.76 - 0.83)	44.7	-11.5	0
Eosinophilic granulomatosis with polyangiitis (Churg-Strauss syndrome)	4.25% (3.05 - 5.74%)	99.9975% (99.9968 - 99.9982%)	2.10% (1.40 - 3.13%)	6.02% (4.07 - 8.82%)	1,733 (1,147 - 2,619)	0.96 (0.94 - 0.97)	15	-14	39
Common variable immunodeficiency	9.67% (7.76 - 11.86%)	99.9966% (99.9957 - 99.9974%)	10.35% (7.77 - 13.67%)	8.76% (6.54 - 11.63%)	2,886 (2,105 - 3,958)	0.90 (0.88 - 0.92)	81.7	-11	-6.5
Dermatomyositis	1.28% (0.91 - 1.76%)	99.9861% (99.9844 - 99.9877%)	1.88% (1.35 - 2.61%)	1.04% (0.75 - 1.45%)	93 (66 - 130)	0.99 (0.98 - 0.99)	36.8	-1	9.5
DiGeorge syndrome (22q11 deletion)	22.65% (20.15 - 25.30%)	99.9690% (99.9665 - 99.9714%)	13.30% (11.80 - 14.96%)	3.29% (2.88 - 3.75%)	730 (637 - 837)	0.77 (0.75 - 0.80)	32.3	-24	5
Duchenne muscular dystrophy	5.95% (4.39 - 7.86%)	99.9949% (99.9938 - 99.9958%)	5.30% (3.83 - 7.30%)	3.84% (2.76 - 5.32%)	1,167 (830 - 1,641)	0.94 (0.92 - 0.96)	50	-3	2.5
Eosinophilic oesophagitis (v1)	0.24% (0.11 - 0.44%)	99.9965% (99.9956 - 99.9973%)	0.44% (0.23 - 0.85%)	1.10% (0.57 - 2.10%)	68 (35 - 131)	1.00 (1.00 - 1.00)	80	-10	-5
Eosinophilic oesophagitis (v2)	6.73% (5.99 - 7.53%)	99.9462% (99.9429 - 99.9494%)	0.81% (0.71 - 0.92%)	2.01% (1.77 - 2.27%)	125 (110 - 142)	0.93 (0.93 - 0.94)	89.1	-14	-9.5
Fibrodysplasia ossificans progressiva	0.00% (0.00 - 1.60%)	99.9999% (99.9996 - 100.0000%)	0.00% (0.00 - 84.19%)	0.00% (0.00 - 84.19%)	0 (Not estimable)	1.00 (1.00 - 1.00)	-	N/A	N/A
Gaucher's disease	6.09% (2.48 - 12.14%)	99.9945% (99.9934 - 99.9955%)	2.18% (1.05 - 4.48%)	0.50% (0.24 - 1.04%)	1,117 (532 - 2,346)	0.94 (0.90 - 0.98)	42.9	-170	12
Good syndrome	100.00% (2.50 - 100.00%)	99.9985% (99.9979 - 99.9990%)	11.76% (8.53 - 16.02%)	0.26% (0.18 - 0.37%)	66,667 (46,612 - 95,349)	0.0 (Not estimable)	100	0	0
Hereditary angioedema	1.21% (0.71 - 1.93%)	99.9903% (99.9889 - 99.9917%)	0.40% (0.24 - 0.65%)	0.69% (0.42 - 1.12%)	125 (76 - 205)	0.99 (0.98 - 0.99)	11.8	-54	51
Hereditary haemorrhagic telangiectasia	1.21% (0.79 - 1.76%)	99.9994% (99.9990 - 99.9997%)	17.57% (9.72 - 29.67%)	14.39% (7.83 - 24.96%)	2,011 (1,016 - 3,980)	0.99 (0.98 - 0.99)	12	-3	55
Hypophosphatasia (v1)	2.37% (0.77 - 5.44%)	99.9509% (99.9477 - 99.9539%)	0.75% (0.32 - 1.77%)	0.04% (0.02 - 0.10%)	48 (20 - 115)	0.98 (0.96 - 1.00)	20	-1	30

Algorithm	Sensitivity	Specificity	PPV (adjusted by literature prevalence)	PPV (adjusted by coded prevalence)	Positive likelihood ratio	Negative likelihood ratio	Percentage flagged pre-dx	Median months flagged relative to dx (of those flagged before dx)	Median months flagged relative to dx (of all flagged patients)
Hypophosphatasia (v2)	0.47% (0.01 - 2.61%)	99.9979% (99.9972 - 99.9985%)	3.42% (0.49 - 20.40%)	0.20% (0.03 - 1.41%)	226 (31 - 1,632)	1.00 (0.99 - 1.00)	0	N/A	1
Myotonic dystrophy (v1)	1.68% (1.18 - 2.31%)	99.9946% (99.9935 - 99.9956%)	6.70% (4.70 - 9.46%)	2.54% (1.76 - 3.66%)	314 (215 - 456)	0.98 (0.98 - 0.99)	36.1	-17	30.5
Myotonic dystrophy (v2)	1.03% (0.64 - 1.55%)	99.9981% (99.9974 - 99.9987%)	11.00% (6.82 - 17.26%)	4.30% (2.59 - 7.04%)	540 (320 - 911)	0.99 (0.99 - 0.99)	27.3	-20	36.5
Narcolepsy	3.05% (2.53 - 3.65%)	99.9996% (99.9993 - 99.9999%)	79.44% (64.33 - 89.23%)	56.24% (37.49 - 73.36%)	8,721 (4,069 - 18,687)	0.97 (0.96 - 0.97)	24.6	-19	2
Niemann-Pick disease, type C	27.78% (9.69 - 53.48%)	99.9928% (99.9915 - 99.9939%)	2.79% (1.32 - 5.80%)	0.27% (0.13 - 0.57%)	3,858 (1,800 - 8,271)	0.72 (0.54 - 0.96)	40	-21.5	0
Osteogenesis imperfecta	1.29% (0.82 - 1.93%)	99.9787% (99.9766 - 99.9807%)	0.35% (0.23 - 0.54%)	0.45% (0.30 - 0.68%)	61 (40 - 92)	0.99 (0.98 - 0.99)	17.4	-21.5	24
Peutz-Jeghers syndrome	1.89% (0.76 - 3.86%)	100.0000% (99.9998 - 100.0000%)	100.00% (59.04 - 100.00%)	100.00% (59.04 - 100.00%)	Inf (Not estimable)	0.98 (0.97 - 1.00)	0	N/A	272
Paroxysmal nocturnal haemoglobinuria	34.95% (28.12 - 42.27%)	99.9771% (99.9749 - 99.9791%)	5.49% (4.47 - 6.73%)	1.24% (1.00 - 1.54%)	1,526 (1,229 - 1,895)	0.65 (0.59 - 0.72)	72.3	-46	-14
PTEN hamartoma tumour syndrome (v1)	8.37% (5.19 - 12.63%)	99.9949% (99.9939 - 99.9959%)	0.66% (0.42 - 1.04%)	1.53% (0.97 - 2.41%)	1,657 (1,043 - 2,632)	0.92 (0.88 - 0.95)	45	-12	12
PTEN hamartoma tumour syndrome (v2)	3.35% (1.46 - 6.49%)	99.9994% (99.9990 - 99.9997%)	2.18% (0.91 - 5.13%)	4.97% (2.11 - 11.25%)	5,579 (2,301 - 13,525)	0.97 (0.94 - 0.99)	25	-136.5	40
Prader-Willi syndrome	2.96% (1.95 - 4.31%)	99.9990% (99.9985 - 99.9994%)	16.51% (9.98 - 26.08%)	9.25% (5.41 - 15.40%)	2,965 (1,661 - 5,290)	0.97 (0.96 - 0.98)	17.4	-36.5	41
SAPHO syndrome	0.67% (0.08 - 2.39%)	99.9968% (99.9960 - 99.9976%)	2.07% (0.52 - 7.93%)	0.25% (0.06 - 0.99%)	212 (52 - 861)	0.99 (0.98 - 1.00)	100	-105.5	-105.5
Sturge-Weber syndrome	13.89% (11.11 - 17.08%)	99.9974% (99.9966 - 99.9981%)	16.02% (11.90 - 21.21%)	10.36% (7.57 - 14.03%)	5,449 (3,860 - 7,691)	0.86 (0.83 - 0.89)	15.7	-4	12.5
Tuberous sclerosis	15.02% (12.97 - 17.27%)	99.9962% (99.9953 - 99.9971%)	16.69% (13.31 - 20.72%)	15.55% (12.36 - 19.37%)	4,006 (3,069 - 5,228)	0.85 (0.83 - 0.87)	10.6	-12	105
Turner syndrome	5.26% (4.41 - 6.22%)	99.9925% (99.9913 - 99.9937%)	15.01% (12.28 - 18.22%)	6.29% (5.05 - 7.81%)	706 (560 - 891)	0.95 (0.94 - 0.96)	30.7	-5	27
Williams syndrome	18.78% (15.63 - 22.27%)	99.9965% (99.9956 - 99.9973%)	42.00% (35.09 - 49.24%)	10.67% (8.18 - 13.79%)	5,444 (4,065 - 7,292)	0.81 (0.78 - 0.85)	20	-15	12.5
Wilson's disease (v1)	0.23% (0.01 - 1.26%)	99.9938% (99.9926 - 99.9948%)	0.06% (0.01 - 0.40%)	0.06% (0.01 - 0.45%)	37 (5 - 261)	1.00 (0.99 - 1.00)	0	N/A	163
Wilson's disease (v2)	0.68% (0.14 - 1.98%)	99.9995% (99.9991 - 99.9998%)	2.29% (0.63 - 7.94%)	2.55% (0.71 - 8.78%)	1,512 (411 - 5,565)	0.99 (0.99 - 1.00)	0	N/A	278
X-linked agammaglobulinaemia	1.82% (0.22 - 6.41%)	99.9977% (99.9970 - 99.9984%)	0.18% (0.04 - 0.73%)	0.35% (0.09 - 1.40%)	808 (198 - 3,290)	0.98 (0.96 - 1.01)	50	-125	-5
X-linked hypophosphataemia (v1)	19.50% (14.70 - 25.08%)	99.9972% (99.9964 - 99.9979%)	8.88% (6.33 - 12.33%)	6.11% (4.32 - 8.59%)	6,965 (4,827 - 10,049)	0.81 (0.76 - 0.86)	23.4	-4	32
X-linked hypophosphataemia (v2)	4.15% (2.01 - 7.50%)	99.9995% (99.9991 - 99.9998%)	10.41% (4.65 - 21.67%)	7.20% (3.15 - 15.59%)	8,299 (3,486 - 19,757)	0.96 (0.93 - 0.98)	0	N/A	95
X-linked hypophosphataemia (v3)	14.11% (9.97 - 19.15%)	99.9880% (99.9864 - 99.9895%)	1.62% (1.16 - 2.25%)	1.09% (0.78 - 1.51%)	1,176 (840 - 1,646)	0.86 (0.82 - 0.90)	9.1	-4	79
