# Supplementary material for: Evaluating algorithmic approaches to rare disease case-finding: a retrospective validation study using electronic health records
Source: Orphanet J Rare Dis. 2026 Feb 4;21:120. doi: 10.1186/s13023-026-04240-6 (PMC13041464; doi:10.1186/s13023-026-04240-6)

Additional file 4

Figure S1: Algorithm performance by specificity

Algorithm performance by specificity, i.e. the proportion of controls correctly not flagged by the algorithm, including Alpha-1-antitrypsin deficiency (v2).

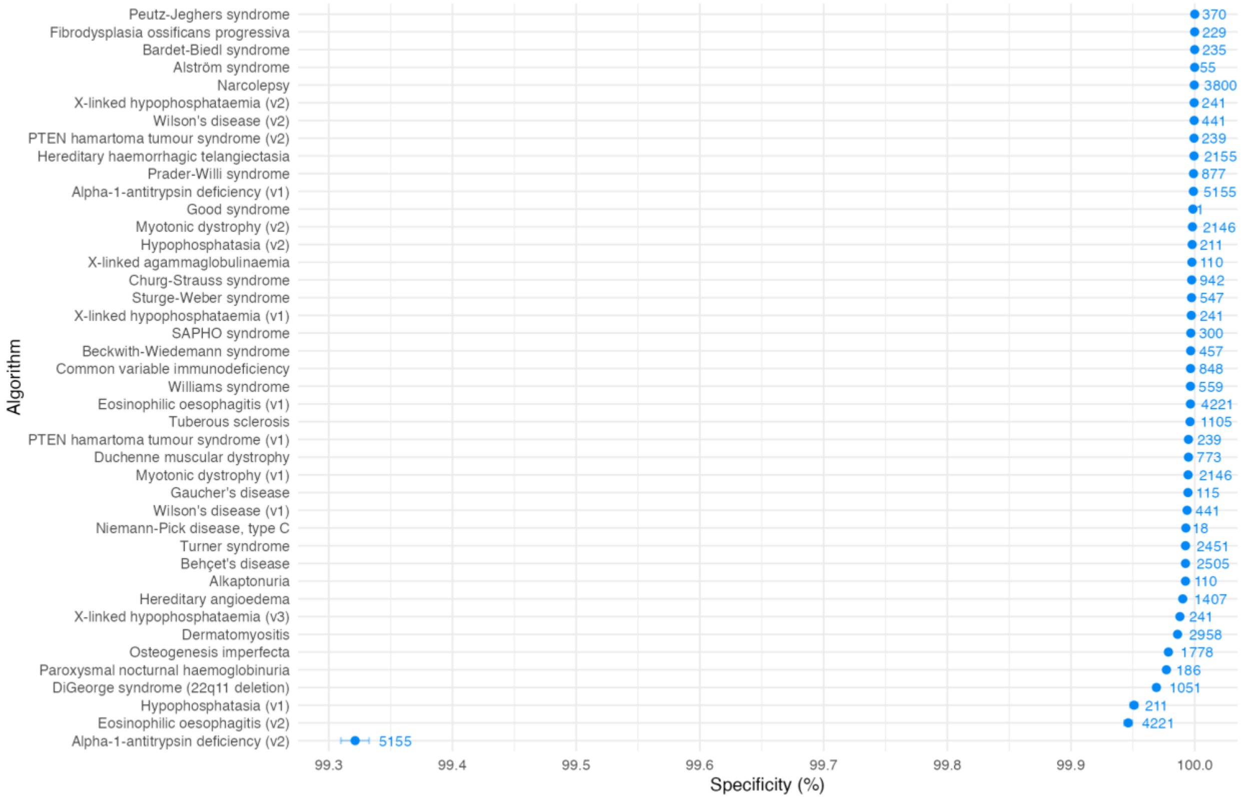

Figure S2: Flag rate

Algorithm performance by expected flag rate per 100,000, including Alpha-1-antitrypsin deficiency (v2). The flag rate is the number of patients expected to be flagged for review by the algorithm.

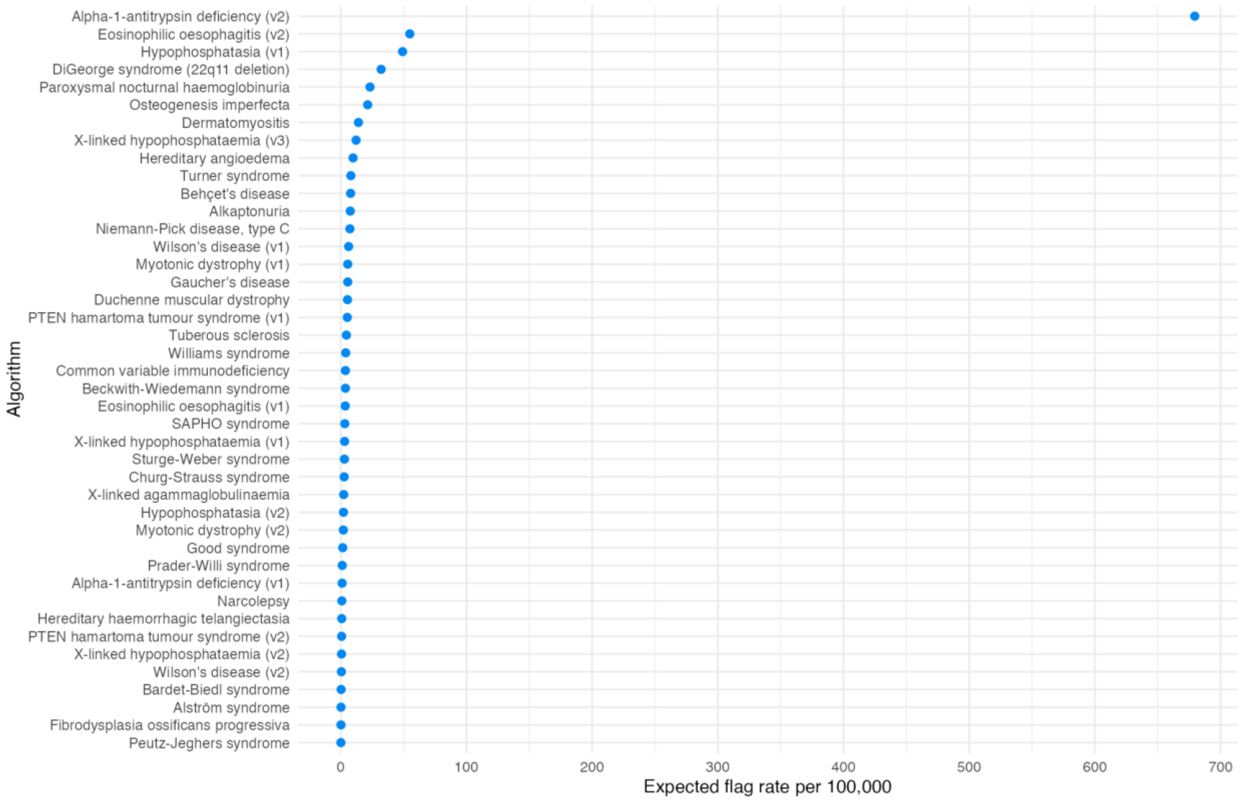

Supplement: Supplementary file 4 — Supplementary Material 4 [file 13023_2026_4240_MOESM4_ESM.pdf]
